# miR-21 deficiency inhibits osteoclast function and prevents bone loss in mice

**DOI:** 10.1038/srep43191

**Published:** 2017-02-27

**Authors:** Cheng-Hu Hu, Bing-Dong Sui, Fang-Ying Du, Yi Shuai, Chen-Xi Zheng, Pan Zhao, Xiao-Rui Yu, Yan Jin

**Affiliations:** 1 Department of Biochemistry and Molecular Biology, School of Basic Medical Sciences, Xi’an Jiaotong University Health Science Center, Xi’an, Shaanxi 710061, China; 2 State Key Laboratory of Military Stomatology & National Clinical Research Center for Oral Diseases & Shaanxi International Joint Research Center for Oral Diseases, Center for Tissue Engineering, Fourth Military Medical University, Xi’an, Shaanxi 710032, China; 3 Xi’an Institute of Tissue Engineering and Regenerative Medicine, Xi’an, Shaanxi 710032, China

## Abstract

MicroRNAs emerge as critical post-transcriptional regulators in bone metabolism. We have previously reported *in vitro* that miR-21 promotes osteogenesis, while studies have also revealed miR-21 as a regulator of osteoclastogenesis and a promoter of osteoclast differentiation *in vitro*. However, *in vivo* data are still lacking in identifying skeletal function of miR-21, particularly its effects on osteoporosis. Here, using miR-21 knockout (miR-21^−/−^) mice, we investigated effects of miR-21 on bone development, bone remodeling and bone loss. Unexpectedly, miR-21^−/−^ mice demonstrated normal skeletal phenotype in development and maintained osteoblastogenesis *in vivo*. Besides, miR-21^−/−^ mice showed increased receptor activator of nuclear factor κB ligand (RANKL) and decreased osteoprotegerin (OPG) through miR-21 targeting Sprouty 1 (Spry1). Nevertheless, interestingly, miR-21 deficiency promoted trabecular bone mass accrual physiologically. Furthermore, in pathological states, the protection of bone mass was prominent in miR-21^−/−^ mice. These skeletal effects were attributed to inhibition of bone resorption and osteoclast function by miR-21 deficiency through miR-21 targeting programmed cell death 4 (PDCD4), despite the existence of RANKL. As far as we know, this is the first *in vivo* evidence of a pro-osteoclastic microRNA. Together, these findings clarified function of miR-21 in bone metabolism, particularly uncovering osteo-protective potential of miR-21 inactivation in osteoporosis.

MicroRNAs post-transcriptionally modulate osteoblastogenesis and osteoclastogenesis and are emerging as critical regulators in bone homeostasis and diseases^1^,^2^. We and others have previously reported *in vitro* that miR-21 promoted osteogenesis of bone marrow mesenchymal stem cells (BMMSCs) by regulating downstream targets including Sprouty 1 (Spry1) and Sprouty 2 (Spry2)^3^,^4^. Previous *in vitro* studies have also revealed pro-osteoclastic function of miR-21 through regulating programmed cell death 4 (PDCD4)^5^, a functional target of miR-21^6^, or by targeting Fas ligand (FasL)^7^. In addition, it has been reported that miR-21 could regulate receptor activator of nuclear factor κB ligand (RANKL) and osteoprotegerin (OPG), the key osteoblastic mediators of osteoclastogenesis^8^,^9^, in multiple myeloma-derived BMMSCs *in vitro*^10^. Interesting questions remain to be answered are that which effect predominates physiologically *in vivo*, and that whether miR-21 regulates skeletal phenotypes in both physiological and pathological states.

Studies have applied targeted delivery of specific antagomirs to modulate and detect skeletal effects of individual microRNAs^11^,^12^,^13^. *In vivo* function of microRNAs in bone could be further uncovered based on gene-manipulated mouse models^13^,^14^,^15^. In osteoporosis, as far as we know, participations of only miR-188 and miR-34a have been revealed in transgenic mice, respectively suppressing osteogenic differentiation of BMMSCs in age-related bone loss and inhibiting osteoclastogenesis in ovariectomy (OVX)-induced osteopenia^13^,^14^. Given *in vitro* promotive effects of miR-21 in osteogenesis and osteoclast differentiation^3^,^5^, elucidating in knockout mice the skeletal function of miR-21, particularly in the development of osteoporosis, is in an urgent need.

In this study, we surprisingly discovered that miR-21 knockout (miR-21^−/−^) mice demonstrated normal skeletal phenotype in development. However, postnatally, miR-21 deficiency promoted trabecular bone mass accrual and prevented bone loss induced by OVX and during aging. Moreover, we demonstrated that these skeletal effects were attributed to inhibited bone resorption and osteoclast function in mice lacking miR-21. Thus, our results clarified physiological and pathophysiological function of miR-21 in the bone metabolism and provided first *in vivo* evidence of a pro-osteoclastic microRNA.

## Results

### miR-21^−/−^ mice demonstrate normal skeletal phenotype in development

miR-21^−/−^ mice were sourced directly from the Jackson Laboratory, and the deficiency of miR-21 was further confirmed systemically (Supplementary Fig. S1a) and in bone (Supplementary Fig. S1b) without affecting the neighborhood of miR-21 gene loci, the gene *Vacuole membrane protein-1 (VMP1*) (Supplementary Fig. S1c,d)^16^,^17^.

**Figure 1:**
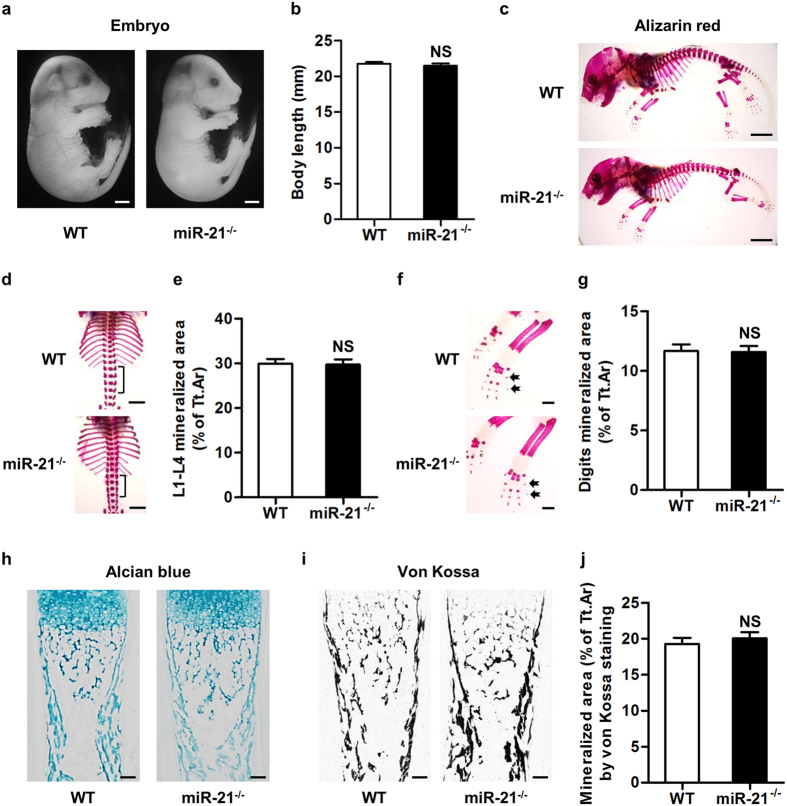
miR-21^−/−^ mice demonstrate normal skeletal phenotype in development. (**a**) miR-21^−/−^ embryos appeared morphologically normal at E18. Bars: 2 mm. (**b**) No significant difference was detected in body length between WT and miR-21^−/−^ embryos at E18. (**c**) Alizarin red staining revealed similar skeletal development in WT and miR-21^−/−^ embryos at E18. Bars: 5 mm. (**d**,**e**) Normal mineralization of miR-21^−/−^ embryos in ribs, thoracic spines and lumbar spines. Black brackets indicate L1-L4 spines analyzed. Tt.Ar, total area. Bars: 2.5 mm. (**f**,**g**) Normal mineralization of miR-21^−/−^ embryos in radius, ulna, carpus and digits. Black arrows indicate representative digits analyzed. Tt.Ar, total area. Bars: 1 mm. (**h**) Alcian blue staining of tibia histological sections at E18 showed comparable cartilaginous remnants in WT and miR-21^−/−^ embryos. Bars: 100 μm. (**i**,**j**) Von Kossa staining of histological sections at E18 demonstrated extensive matrix mineralization in tibia of both WT and miR-21^−/−^ embryos. Tt.Ar, total area. Bars: 100 μm. Data represents mean ± standard errors of the mean. n = 6/genotype. Statistical significance was evaluated by two-tailed Student’s t test. NS, not significant (*P* > 0.05).

To clarify skeletal effects of miR-21, we firstly investigated skeletal phenotypes of miR-21^−/−^ embryos. To our surprise, miR-21^−/−^ embryos at E18 appeared morphologically normal (Fig. 1a), with comparable body length to those of WT (Fig. 1b). Moreover, alizarin red staining at E18 demonstrated similar overall skeletal mineralization in WT and miR-21^−/−^ embryos (Fig. 1c). Further observations showed that mineralized area in ribs, thoracic spines and lumbar spines of miR-21^−/−^ embryos were as much as those of WT embryos (Fig. 1d). Quantitative analysis on L1-L4 mineralization did not detect significant difference (Fig. 1e). Also, WT and miR-21^−/−^ embryos showed paralleled mineralized area in radius, ulna, carpus and digits (Fig. 1f,g).

Bone development is determined primarily by the bone modeling process, in which endochondral ossification is the mechanism whereby long bones are formed^18^,^19^. To further dissect the skeletal function of miR-21 in embryos, we analyzed the cartilage remnants and the ossification that are key to bone mass accrual, as previously reported^15^,^20^. Histological analyses illustrated comparable cartilaginous remnants, hypertrophic chondrocytes (Fig. 1h) and mineralization in tibia (Fig. 1i,j) of WT and miR-21^−/−^ embryos at E18. These results indicated normal skeletal phenotype in development of miR-21^−/−^ mice.

### miR-21 regulates osteoblastogenesis and maintains bone formation *in vivo*

**Figure 2:**
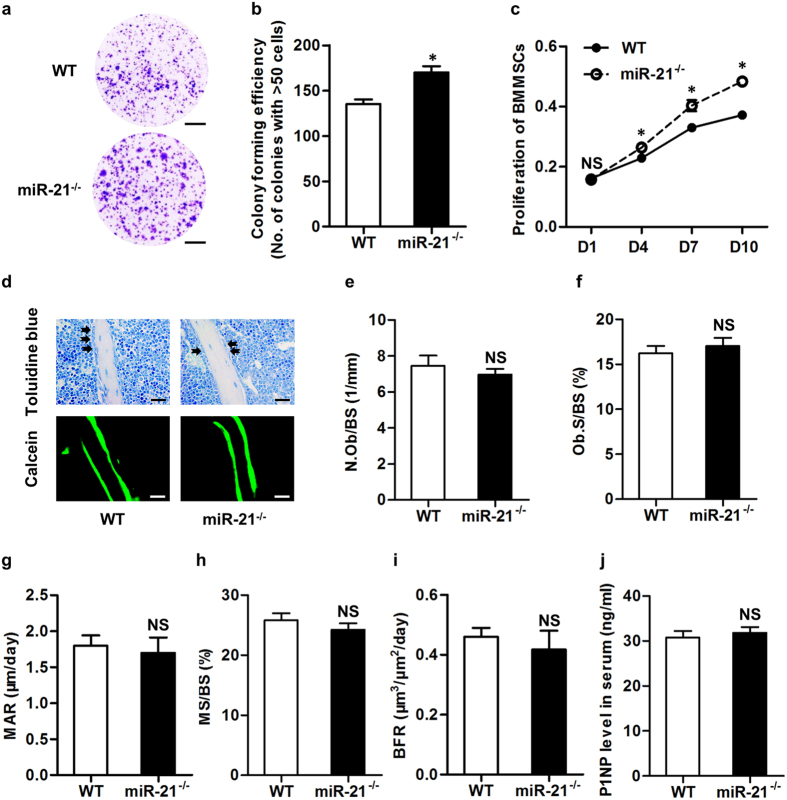
miR-21 regulates osteoblastogenesis and maintains bone formation *in vivo*. (**a**,**b**) Bone marrow mesenchymal stem cells (BMMSCs) derived from miR-21^−/−^ mice showed increased colony forming efficiency. Primary bone marrow cells were isolated from 3-month WT and miR-21^−/−^ mice, seeded at 1 × 10^5^ cells/cm^2^, cultured for 14 days, and stained with crystal violet. Colonies with over 50 cells were taken into account. Bars: 1 cm. (**c**) BMMSCs derived from miR-21^−/−^ mice showed increased proliferation rate. 1^st^ passaged BMMSCs isolated from 3-month WT and miR-21^−/−^ mice were seeded at 2 × 10^3^ cells/well in 96-well plates. Cell viability was determined by methyl thiazolyl tetrazolium (MTT) assay at indicated time points. (**d**) Toluidine blue staining (top) and calcein labeling (bottom) in histological sections of 3-month WT and miR-21^−/−^ mice. Mice accepted double intraperitoneal injection of 20 mg/kg calcein at 16 days and 2 days prior to sacrifice. After sacrifice, tibiae were decalcified, embedded in paraffin, sectioned, and stained for toluidine blue. Femora were embedded in methyl methacrylate without decalcification, sectioned, and observed by a fluorescence microscope on the endosteum. Black arrows indicate osteoblasts analyzed on trabecular bone surfaces. Bars (top): 25 μm; Bars (bottom): 100 μm. (**e**,**f**) Corresponding parameters of toluidine blue staining showed comparable osteoblastogenesis in WT and miR-21^−/−^ mice. N.Ob/BS, number of osteoblasts per bone surface (**e**). Ob.S/BS, osteoblast surface per bone surface (**f**). (**g**–**i**) Corresponding parameters detected by calcein labeling showed comparable bone formation in WT and miR-21^−/−^ mice. MAR, mineral apposition rate (**g**). MS/BS, mineralized surface per bone surface (**h**). BFR, bone formation rate (**i**). (**j**) No significant difference was detected by the enzyme-linked immunosorbent assay (ELISA) on the concentrations of bone formation marker in serum of 3-month WT and miR-21^−/−^ mice. P1NP, procollagen 1 N-terminal peptide. Data represents mean ± standard errors of the mean. n = 6/genotype. Statistical significance was evaluated by two-tailed Student’s t test. *P < 0.05. NS, not significant (*P* > 0.05).

Given the normal skeletal phenotype of miR-21^−/−^ embryos and the role of miR-21 as an osteogenesis promoter of BMMSCs *in vitro*^3^, we next examined effects of miR-21 in characteristics of BMMSCs *ex vivo*. We confirmed reduction of osteogenic differentiation of *ex vivo* miR-21^−/−^ BMMSCs that may be attributed to *SPRY1* up-regulation (Supplementary Fig. S2). However, we further discovered that primary miR-21^−/−^ BMMSCs showed increased colony forming efficiency (Fig. 2a,b), and that miR-21^−/−^ BMMSCs continued to show increased proliferation rate during passages (Fig. 2c). These results suggested that miR-21 inhibited colony formation and proliferation of BMMSCs despite promotion on osteogenesis.

We next investigated effects of miR-21 deficiency on osteoblastogenesis *in vivo*, by using toluidine blue staining for osteoblasts and calcein labeling for bone formation (Fig. 2d). Surprisingly, we revealed that number and surface of osteoblasts per bone surface were not significantly different between 3-month WT and miR-21^−/−^ mice (Fig. 2e,f). We also revealed that bone formation parameters were comparable in WT and miR-21^−/−^ mice (Fig. 2g,h). Furthermore, the level of a bone formation marker in serum, procollagen 1 N-terminal peptide (P1NP), in miR-21^−/−^ mice was paralleled with that in WT mice. These findings highlighted that miR-21 maintained bone formation and osteoblastogenesis *in vivo*.

### miR-21 deficiency promotes trabecular bone mass accrual postnatally

**Figure 3:**
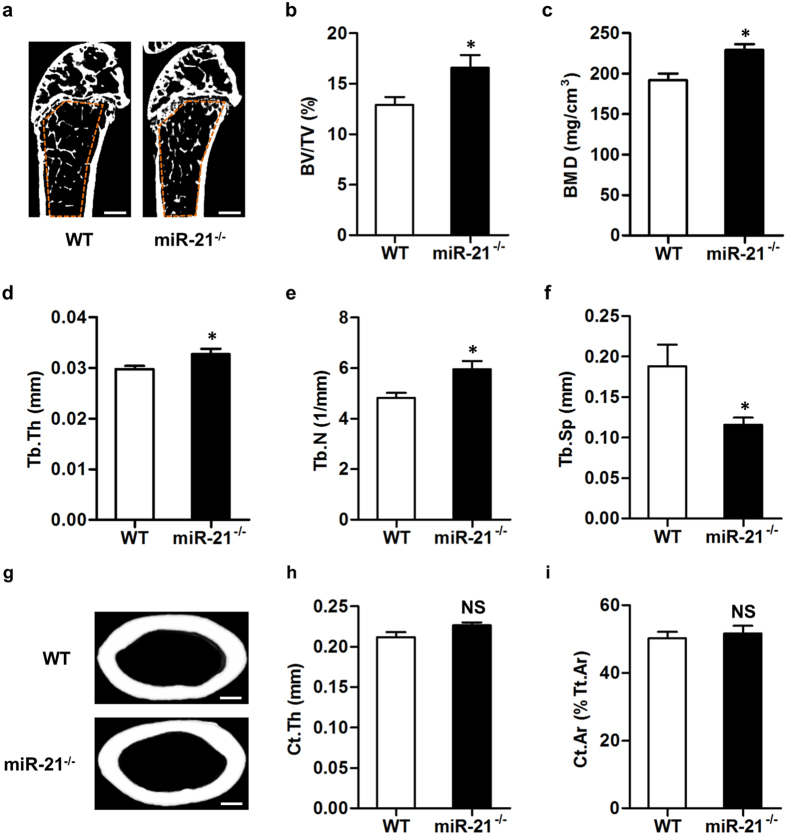
miR-21^−/−^ mice show increased trabecular bone mass accrual postnatally. (**a**) Representative micro-CT images demonstrating bone phenotypes of 3-month WT and miR-21^−/−^ mice. Orange frames indicate the region of interest analyzed for trabecular bone mass in the distal femoral metaphysis. Bars: 500 μm. (**b**–**f**) Corresponding parameters showed high trabecular bone mass phenotype of 3-month miR-21^−/−^ mice. BV/TV, bone volume per tissue volume (**b**). BMD, bone mineral density (**c**). Tb.Th, trabecular thickness (**d**). Tb.N, trabecular number (**e**). Tb.Sp, trabecular separation (**f**). (**g**) Representative cortical bone images in the midshaft of femora of 3-month WT and miR-21^−/−^ mice. Bars: 500 μm. (**h, i**) Corresponding parameters showed normal cortical bone phenotype of 3-month miR-21^−/−^ mice. Ct.Th, cortical thickness (**h**). Ct.Ar, cortical area. Tt.Ar, total area (**i**). Data represents mean ± standard errors of the mean. n = 6/genotype. Statistical significance was evaluated by two-tailed Student’s t test. *P < 0.05. NS, not significant (*P* > 0.05).

To further study the skeletal phenotype of miR-21^−/−^ mice, we separately analyzed trabecular and cortical bone mass of WT and miR-21^−/−^ mice using the micro-CT system. As shown, miR-21^−/−^ mice had a slightly increased trabecular bone mass compared to WT mice at 3-month old (Fig. 3a). Quantifications on the trabecular bone volume (Fig. 3b) and bone mineral density (BMD) (Fig. 3c) confirmed that miR-21 deficiency promotes trabecular bone mass accrual. These changes were attributed to increases in the thickness and number of the trabecular bone (Fig. 3d,e), and a decrease in the separation of the trabecular bone (Fig. 3f), as shown by trabecular bone parameters. However, we did not detect differences in cortical bone mass between WT and miR-21^−/−^ mice (Fig. 3g), provided the thickness and area of the cortical bone were comparable (Fig. 3h,i). Besides, WT and miR-21^−/−^ mice showed similar body composition (Supplementary Table S1). These findings suggested that miR-21 functioned to suppress trabecular bone mass accrual postnatally.

### miR-21 controls osteoclastogenesis by regulating RANKL and OPG

**Figure 4:**
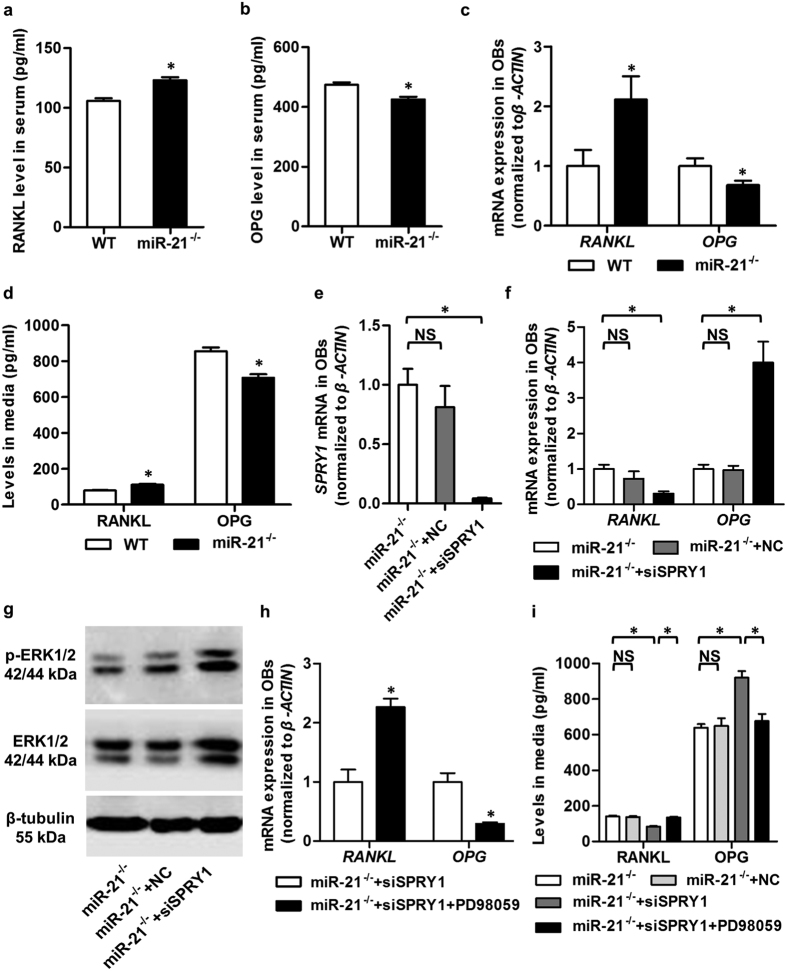
miR-21 regulates receptor activator of nuclear factor κB ligand (RANKL) and osteoprotegerin (OPG) by targeting Sprouty 1 (Spry1) to modulate extracellular signal-regulated kinase (ERK) signaling in osteoblasts (OBs). (**a**,**b**) Enzyme-linked immunosorbent assay (ELISA) detection of serum concentrations of RANKL (**a**) and OPG (**b**). Increased RANKL and decreased OPG were detected in miR-21^−/−^ mice, suggest that the increased bone mass in miR-21^−/−^ mice was not attributed to RANKL or OPG changes. (**c**) Quantitative real-time polymerase chain reaction (qRT-PCR) analysis demonstrated up-regulated mRNA level of *RANKL* and down-regulated mRNA level of *OPG* in OBs from 3-month WT and miR-21^−/−^ mice. (**d**) Concentrations of RANKL and OPG were determined by ELISA in culture media of OBs. miR-21^−/−^ OBs showed increased RANKL secretion and decreased OPG secretion. (**e**) qRT-PCR analysis of miR-21^−/−^ OBs demonstrated down-regulation of mRNA level of *SPRY1* by small interfering RNA. siSPRY1, small interfering RNA for SPRY1. NC, negative control of siSPRY1. (**f**) qRT-PCR analysis demonstrated suppression of mRNA level of *RANKL* and rescue of mRNA level of *OPG* in miR-21^−/−^ OBs by siSPRY1. (**g**) Western blot analysis of miR-21^−/−^ OBs. siSPRY1 stimulated ERK signaling at both total and phosphorylated protein expression levels. Cropped blots are displayed with only brightness adjusted equally across the entire images. (**h**) qRT-PCR analysis demonstrated increased mRNA level of *RANKL* and decreased mRNA level of *OPG* in SPRY1-down-regulated miR-21^−/−^ OBs by PD98059, an ERK inhibitor. (**i**) Concentrations of RANKL and OPG were determined by ELISA in culture media of miR-21^−/−^ OBs. Data demonstrated that miR-21 regulated RANKL and OPG by targeting Spry1 to regulate ERK signaling. Data represents mean ± standard errors of the mean. n = 6/genotype (**a**–**d**), n = 3/group (**e**–**h**) and n = 4/group (**i**). Statistical significance was evaluated by two-tailed Student’s t test for two-group comparison, and one way analysis of variation (ANOVA) with Newman-Keuls post-hoc tests for multiple comparisons. *P < 0.05. NS, not significant (*P* > 0.05).

We next examined whether miR-21 regulates osteoclastogenesis to promote trabecular bone mass. The key osteoblastic mediators of osteoclastogenesis, RANKL^8^ and OPG^9^, were analyzed *in vivo*. Unexpectedly, enzyme-linked immunosorbent assay (ELISA) of serological levels demonstrated promoted RANKL and suppressed OPG by miR-21 deficiency (Fig. 4a,b), indicating stimulation of osteoclastogenesis. These effects were confirmed by corresponding changes in mRNA expression levels of *RANKL* and *OPG* in osteoblasts (Fig. 4c), and in their secretion of RANKL and OPG into culture media (Fig. 4d). Therefore, although we discovered modulatory effects of miR-21 on RANKL and OPG, these findings suggested that the increased postnatal trabecular bone mass in miR-21^−/−^ mice was not attributed to changes of osteoclastogenesis.

To dissect the mechanism underlying miR-21 regulating RANKL and OPG in osteoblastic lineage cells, we tested if our previously established miR-21 target in BMMSCs, Spry1^3^, is a regulator of both RANKL and OPG. Given that *SPRY1* was up-regulated in miR-21-deficient osteoblastic lineage cells (Supplementary Fig. S2c,d), we applied small interfering RNA (siRNA) for *SPRY1* (siSPRY1) in miR-21-deficient osteoblasts. Quantitative real-time polymerase chain reaction (qRT-PCR) analysis demonstrated successful down-regulation of *SPRY1* mRNA level by siSPRY1, but not its negative control (NC) (Fig. 4e). Furthermore, siSPRY1 reduced *RANKL* and rescued *OPG* expression in miR-21-deficient osteoblasts (Fig. 4f), suggesting Spry1 is a functional target of miR-21 in regulating osteoclastogenesis.

We further explored the molecular mediator(s) downstream Spry1 to regulate RANKL and OPG, for Spry1 binding sites were not found in the promotor regions of RANKL and OPG, suggesting indirect modulating manners. Extracellular signal-regulated kinase (ERK) signaling has been reported to inhibit RANKL and promote OPG under mechanical force in fibroblasts^21^, and it has been proved to be regulated by miR-21 targeting Spry1^22^,^23^. We confirmed in this study that siSPRY1 induced both ERK1/2 and p-ERK1/2 in miR-21-deficient osteoblasts (Fig. 4g). Using a pharmacological ERK inhibitor, PD98059, we further revealed that ERK inhibition could oppose effects of siSPRY1 on *RANKL* and *OPG* expression and moreover, the secretion, in miR-21-deficient osteoblasts (Fig. 4h,i). These findings collectively indicated that miR-21 regulates RANKL and OPG by targeting Spry1 to modulate ERK signaling in osteoblasts.

### miR-21 promotes bone resorption *in vivo* and supports osteoclast function

**Figure 5:**
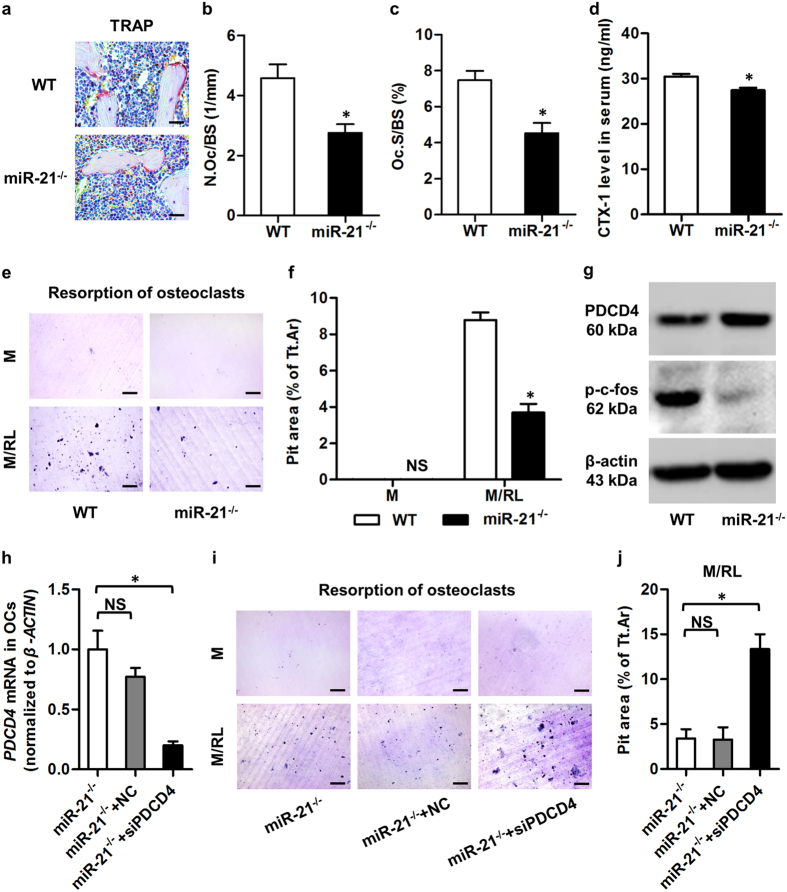
miR-21 promotes bone resorption *in vivo* and controls osteoclastogenesis by targeting programmed cell death 4 (PDCD4). (**a**) Tartrate resistant acid phosphotase (TRAP) staining of the trabecular bone in histological sections of 3-month WT and miR-21^−/−^ mice. Tibiae were decalcified, embedded in paraffin, sectioned, and stained for TRAP. Bars: 25 μm. (**b**,**c**) Corresponding parameters showed inhibited osteoclastogenesis and bone resorption in miR-21^−/−^ mice. N.Oc/BS, number of osteoclasts per bone surface (**b**). Oc.S/BS, osteoclast surface per bone surface (**c**). (**d**) Enzyme-linked immunosorbent assay (ELISA) detection of the serum bone resorption marker of 3-month WT and miR-21^−/−^ mice. miR-21 deficiency inhibited the bone resorption rate. CTX-1, cross linked C-telopeptide of type 1 collagen. (**e**,**f**) Representative images (**e**) and the corresponding parameter (**f**) demonstrated that miR-21^−/−^ osteoclasts (OCs) generated declined resorption pits on dentine slices. Resorption pits were stained with toluidine blue. M, macrophage colony-stimulating factor (M-CSF). RL, receptor activator of nuclear factor κB ligand (RANKL). Tt.Ar, total area. Bars: 100 μm. (**g**) Western blot analysis of mature OCs derived from 3-month WT and miR-21^−/−^ mice. OCs were differentiated with M-CSF and RANKL. miR-21 deficiency promoted the PDCD4 protein level, a functional target of miR-21, which suppressed the phosphorylation level of c-fos. Cropped blots are displayed with only brightness adjusted equally across the entire images. (**h**) Quantitative real-time polymerase chain reaction (qRT-PCR) analysis of miR-21^−/−^ mature OCs demonstrated down-regulation of mRNA level of *PDCD4* by small interfering RNA. OCs were differentiated with M-CSF and RANKL. siPDCD4, small interfering RNA for PDCD4. NC, negative control of siPDCD4. (**i**,**j**) Representative images (**i**) and the corresponding parameter (**j**) demonstrated that down-regulation of PDCD4 rescued resorption capability of miR-21^−/−^ OCs on dentine slices. Resorption pits were stained with toluidine blue. Bars: 100 μm. Data represents mean ± standard errors of the mean. n = 6/genotype (**a**–**g**), n = 3/group (**h**) and n = 4/group (**i**,**j**). Statistical significance was evaluated by two-tailed Student’s t test for two-group comparison, and one way analysis of variation (ANOVA) with Newman-Keuls post-hoc tests for multiple comparisons. *P < 0.05. NS, not significant (*P* > 0.05).

The above data inspired us to further investigate whether the increased bone mass of miR-21^−/−^ mice was directly attributed to impaired bone resorption and osteoclast function. As expected, depicted by tartrate resistant acid phosphotase (TRAP) staining, miR-21^−/−^ mice showed inhibited bone resorption at 3-month old (Fig. 5a), which was attributed to declined parameters of the number and surface of osteoclasts in miR-21^−/−^ mice (Fig. 5b,c). Analyses on the level of serological marker confirmed that miR-21 deficiency reduced the bone resorption rate, as shown by the cross linked C-telopeptide of type 1 collagen (CTX-1) (Fig. 5d) concentration.

To confirm effects of miR-21 in RANKL-induced osteoclast differentiation and activity, TRAP staining and resorption examination were respectively performed. We revealed that despite the existence of RANKL, miR-21 deficiency inhibited osteoclast differentiation, as shown by declined formation of TRAP^+^ multinucleated cells (Supplementary Fig. S3a,b). We further discovered that miR-21 deficiency reduced resorption activity of osteoclasts, as demonstrated by declined resorption pits on dentine slices (Fig. 5e,f).

PDCD4 was previously demonstrated to regulate osteoclast differentiation^5^ and was revealed as a direct target of miR-21^6^. We next uncovered that the effects in miR-21^−/−^ osteoclasts were indeed attributed to an increase of PDCD4 protein level (Fig. 5g), while the mRNA level of *PDCD4* remained unchanged (Supplementary Fig. S3c), confirming miR-21 regulation of PDCD4 expression in osteoclasts at the posttranscriptional level. This targeted regulation of miR-21 on PDCD4 lead to down-regulation of both mRNA expression of *c-FOS* and its protein phosphorylation (p-c-fos) level (Fig. 5g, Supplementary Fig. S3d), which is a critical transcription factor for osteoclastogenesis^5^. In addition, we showed up-regulation of PDCD4 and down-regulation of p-c-fos in bone marrow of miR-21^−/−^ mice (Supplementary Fig. S3e,f).

To further prove the role of PDCD4 in mediating effects of miR-21 on RANKL-induced osteoclast function, we applied siRNA for *PDCD4* (siPDCD4) during miR-21-deficient osteoclast differentiation. qRT-PCR analysis demonstrated successful down-regulation of *PDCD4* mRNA level by siPDCD4, but not its NC (Fig. 5h). Furthermore, under the existence of RANKL, siPDCD4 rescued both differentiation and resorption activity of miR-21-deficient osteoclasts, as shown by recovered formation of TRAP^+^ multinucleated cells (Supplementary Fig. S3g,h) and resorption pits on dentine slices (Fig. 5i,j). In addition, mRNA expression of *c-FOS* was also promoted by siPDCD4 (Supplementary Fig. S3i). These findings suggested PDCD4 is a functional target of miR-21 in supporting osteoclast function, collectively indicating that miR-21 promotes bone resorption *in vivo* through direct control of osteoclast function by targeting PDCD4.

### miR-21 deficiency blocks OVX-induced osteopenia by inhibiting osteoclast function

**Figure 6:**
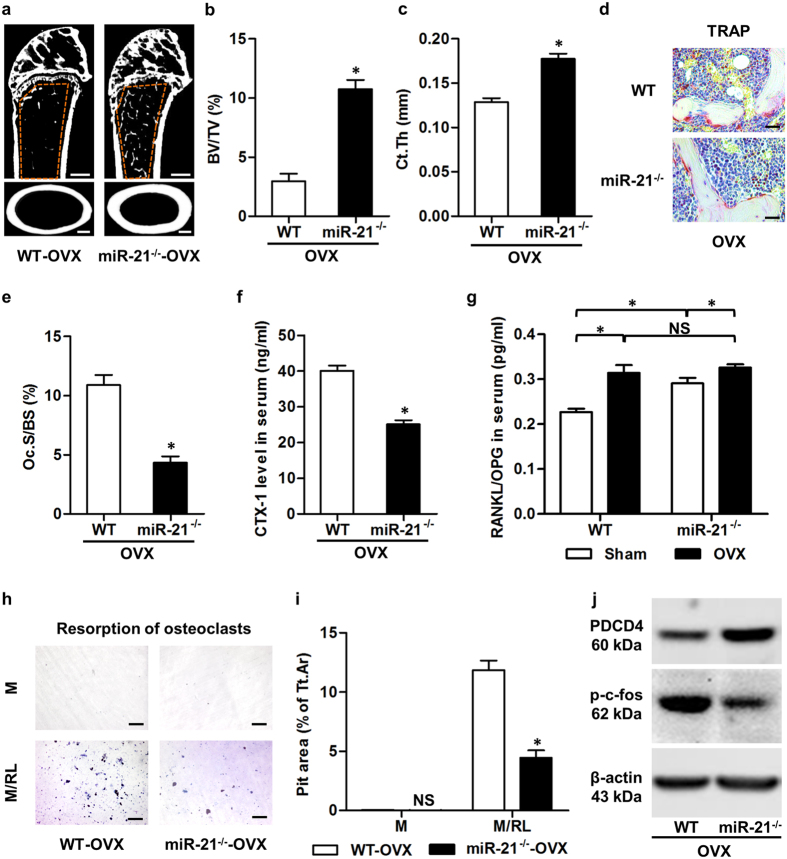
miR-21 deficiency blocks ovariectomy (OVX)-induced osteopenia by inhibiting osteoclastogenesis through targeting programmed cell death 4 (PDCD4). (**a**) Representative micro-CT images demonstrating bone phenotypes of ovariectomized WT and miR-21^−/−^ mice. Mice were sacrificed at 1 month post OVX. Orange frames indicate the region of interest analyzed for trabecular bone mass in the distal femoral metaphysis (up). Cortical bone mass was analyzed in the midshaft of femora (bottom). Bars: 500 μm. (**b**,**c**) Corresponding parameters showed that miR-21 deficiency prevented both trabecular (**b**) and cortical (**c**) bone loss induced by OVX. BV/TV, bone volume per tissue volume. Ct.Th, cortical thickness. (**d**) Tartrate resistant acid phosphotase (TRAP) staining of the trabecular bone in histological sections of ovariectomized WT and miR-21^−/−^ mice. Bars: 25 μm. (**e**,**f**) The corresponding parameter of TRAP and the serum bone resorption marker detected by enzyme-linked immunosorbent assay (ELISA) showed that miR-21 deficiency blocked OVX-induced osteoclastogenesis and bone resorption. Oc.S/BS, osteoclast surface per bone surface (**e**). CTX-1, cross linked C-telopeptide of type 1 collagen (**f**). (**g**) ELISA detection of serum ratio of receptor activator of nuclear factor κB ligand (RANKL) over osteoprotegerin (OPG). No significant difference was detected between ovariectomized WT and miR-21^−/−^ mice. (**h**,**i**) Representative images (**h**) and the corresponding parameter (**i**) demonstrated that miR-21 deficiency blocked OVX-induced resorption activity of osteoclasts (OCs). Bone marrow macrophages (BMMs) were harvested, seeded on dentine slices and cultured. Resorption pits were stained with toluidine blue. M, macrophage colony-stimulating factor (M-CSF). RL, RANKL. Tt.Ar, total area. Bars: 100 μm. (**j**) Western blot analysis of mature osteoclasts derived from ovariectomized WT and miR-21^−/−^ mice. miR-21 deficiency inhibited OVX-induced osteoclastogenesis through promoting the PDCD4 protein level, a functional target of miR-21, which suppressed the phosphorylation level of c-fos. Cropped blots are displayed with only brightness adjusted equally across the entire images. Data represents mean ± standard errors of the mean. n = 6 per group. Statistical significance was evaluated by two-tailed Student’s t test for two-group comparison, and one way analysis of variation (ANOVA) with Newman-Keuls post-hoc tests for multiple comparisons. *P < 0.05. NS, not significant (*P* > 0.05).

We next investigated the pathophysiological role of miR-21 in estrogen deficiency-induced osteoporosis. Micro-CT analysis showed that miR-21 deficiency blocked OVX-induced osteopenia (Fig. 6a), and that both trabecular and cortical bone loss were prevented (Fig. 6b,c). These effects were not attributed to a rescue in osteoblastogenesis or bone formation in miR-21^−/−^ mice (Supplementary Fig. S4a–c). Instead, OVX-induced bone resorption was prevented by miR-21 deficiency (Fig. 6d–f). The RANKL/OPG ratio was not significantly different between ovariectomized WT and miR-21^−/−^ mice (Fig. 6g). However, both differentiation (Supplementary Fig. S4d,e) and resorption activity (Fig. 6h,i) of osteoclasts from ovariectomized miR-21^−/−^ mice were impaired. We further revealed up-regulation of the miR-21 target PDCD4 protein level, which suppressed *c-FOS* and p-c-fos expression in osteoclasts from ovariectomized miR-21^−/−^ mice, compared to those derived from ovariectomized WT mice (Fig. 6j, Supplementary Fig. S4f,g). We also confirmed that miR-21 targeted PDCD4 to modulate p-c-fos *in vivo* after OVX (Supplementary Fig. S4h,i). These findings highlighted that miR-21 deficiency blocks OVX-induced osteopenia by inhibiting osteoclast function through targeting PDCD4.

### miR-21 contributes to age-related osteopenia and bone loss in human

**Figure 7:**
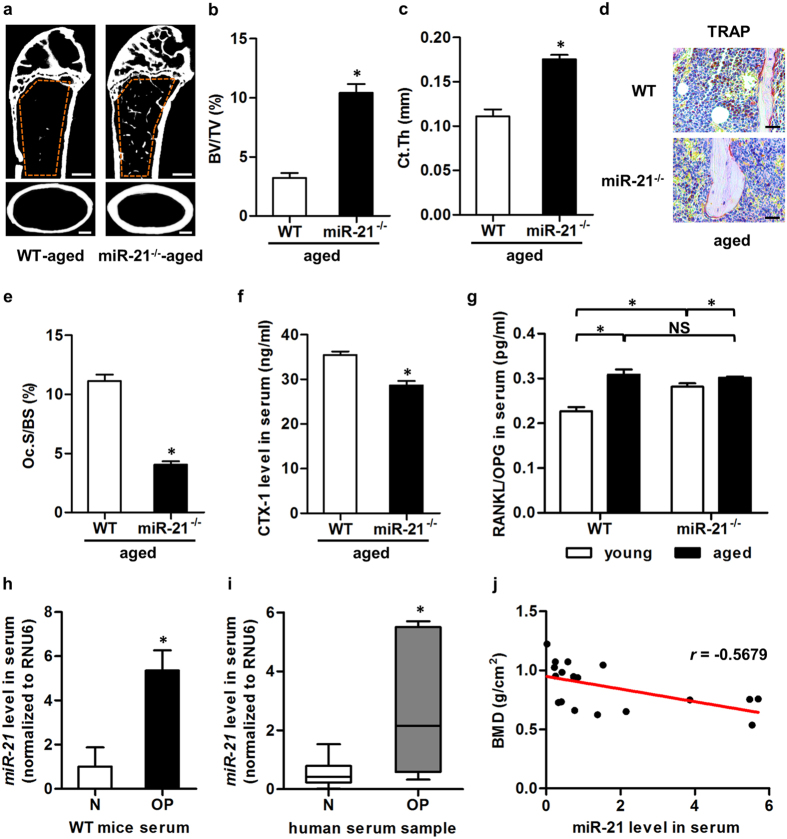
miR-21 contributes to age-related osteopenia and bone loss in human. (**a**) Representative micro-CT images demonstrating bone phenotypes of 16-month WT and miR-21^−/−^ mice. Orange frames indicate the region of interest analyzed for trabecular bone mass in the distal femoral metaphysis (up). Cortical bone mass was analyzed in the midshaft of femora (bottom). Bars: 500 μm. (**b**,**c**) Corresponding parameters showed that miR-21 deficiency prevented age-related trabecular (**b**) and cortical (**c**) bone loss. BV/TV, bone volume per tissue volume. Ct.Th, cortical thickness. (**d**) Tartrate resistant acid phosphotase (TRAP) staining of the trabecular bone of 16-month WT and miR-21^−/−^ mice. Tibiae were decalcified, embedded in paraffin, sectioned, and stained for TRAP. Bars: 25 μm. (**e**,**f**) The corresponding parameter of TRAP and the serum bone resorption marker detected by enzyme-linked immunosorbent assay (ELISA) showed that miR-21 deficiency blocked age-related osteoclastogenesis and bone resorption. Oc.S/BS, osteoclast surface per bone surface (**e**). CTX-1, cross linked C-telopeptide of type 1 collagen (**f**). (**g**) ELISA detection of serum ratio of receptor activator of nuclear factor κB ligand (RANKL) over osteoprotegerin (OPG). No significant difference was detected between 16-month WT and miR-21^−/−^ mice. (**h**) Quantitative real-time polymerase chain reaction (qRT-PCR) analysis demonstrated up-regulated mRNA level of miR-21 in serum of osteoporotic mice. N, normal. OP, osteopenia induced by ovariectomy (OVX). The above data represents mean ± standard errors of the mean. n = 6 per group of mice. Statistical significance was evaluated by two-tailed Student’s t test for two-group comparison, and by one way analysis of variation (ANOVA) followed by Newman-Keuls post-hoc tests for multiple comparisons. *P < 0.05. NS, not significant (*P* > 0.05). (**i**) In osteoporotic human samples, qRT-PCR analysis also detected up-regulated mRNA level of miR-21 in serum. N, healthy donor. OP, donor with postmenopausal osteoporosis. n = 9 per group. Results are given as box plots showing 5th, 50th and 95th percentiles, and minimum to maximum ranges. Two-tailed Mann-Whitney U test was used to determine the significance. *P < 0.05. (**j**) Bone mineral density (BMD) was inversely correlated with miR-21 in human serum. Pearson’s correlation: −0.5679; *p* = 0.0140.

To further determine whether miR-21 contributed to the development of osteoporosis, we examined effects of miR-21 deficiency in age-related osteopenia. Micro-CT analysis showed that miR-21^−/−^ mice did not develop age-related osteopenia (Fig. 7a), and that both trabecular and cortical bone mass were maintained (Fig. 7b,c). Impairments were still detected in osteoblastogenesis and bone formation of aged miR-21^−/−^ mice (Supplementary Fig. S5a–c). However, age-related elevation of bone resorption was prevented by miR-21 deficiency (Fig. 7d–f), which may be attributed to targeted regulation of PDCD4 that lead to p-c-fos up-regulation (Supplementary Fig. S5d,e). In addition, the RANKL/OPG ratio was comparable between aged WT and miR-21^−/−^ mice (Fig. 7g). These results suggested that miR-21 contributed to the development of osteopenia during aging.

To identify the correlation of miR-21 changes with bone loss, we detected serological miR-21 levels in normal and osteoporotic mice and individuals. As depicted, the mean of serological relative miR-21 levels of osteoporotic mice was 4-fold higher compared to that of normal mice, and the difference was statistically significant (Fig. 7h). Similarly in human samples, serological relative miR-21 levels of osteoporotic individuals were significantly higher compared to that of normal individuals (Fig. 7i). Further analysis identified correlation of serological relative miR-21 levels with BMD of human lumbar spine in the development of osteoporosis (Pearson’s correlation: −0.5679; *p* = 0.0140) (Fig. 7j). These findings highlighted skeletal effects of miR-21 in correlation with bone homeostasis.

## Discussion

Critical function of individual microRNAs in bone is emerging to be revealed^13^,^14^,^15^. Previous *in vitro* studies have shown that miR-21 regulates osteoclast differentiation^5^,^7^, osteoclastogenesis^10^ and osteogenesis of BMMSCs^3^,^4^. In the present study, we further discovered *in vivo* the protection of bone mass in miR-21^−/−^ mice that was attributed to an inhibition of osteoclast function. Our results clarified skeletal function of miR-21 and provided first *in vivo* evidence of a pro-osteoclastic microRNA.

microRNAs post-transcriptionally modulate properties of both osteoclastic and osteoblastic lineage cells^1^,^2^. Function of microRNAs in osteoblastogenesis has been well established by numerous *in vitro* reports and several *in vivo* studies^1^,^2^,^11^,^13^,^15^,^24^. However, a few individual microRNAs have been demonstrated to regulate osteoclastogenesis^2^,^5^, among which only miR-34a was uncovered based on transgenic mouse models as a key osteoclast suppressor to confer skeletal protection^14^. Notably, miR-34b/c were revealed to specifically regulate osteoblastogenesis in bone^15^,^24^. This skeletal functional diversity of microRNAs from one family was further supported by findings of miR-21. miR-21 was previously reported as an osteogenesis promoter of BMMSCs by *in vitro* studies^3^,^4^ and in applied researches targeting miR-21 or downstream effectors to promote bone formation^25^,^26^. miR-21 was also documented *in vitro* as a microRNA expression signature of RANKL-induced osteoclast differentiation^5^ and to oppose pro-apoptotic effect of estrogen on mature osteoclasts^7^. In addition, it has been reported that miR-21 could regulate RANKL and OPG, the key osteoblastic mediators of osteoclastogenesis^8^,^9^, in multiple myeloma-derived BMMSCs *in vitro*^10^. Here, we clarified that the function of miR-21 in promoting osteoclast function predominated *in vivo*, despite that it maintained osteoblastogenesis, inhibited RANKL and promoted OPG physiologically. As far as we know, this is the first *in vivo* evidence of a pro-osteoclastic microRNA based on gene-manipulated animal models.

Correlations of microRNA function with bone diseases have just begun to emerge. With the targeted delivery of specific antagomirs to bone cells, inhibition of miR-148a, miR-188 and miR-214 restored bone mass in osteoporotic mice^11^,^12^,^13^. In transgenic mice, miR-188 was further revealed to regulate the switch between osteoblast and adipocyte differentiation in age-related bone loss^13^. Moreover, miR-34a overexpression in mice blocks osteoporosis and osteolytic bone metastasis^14^. In our previous research, we found that miR-21 in BMMSCs was regulated by tumor necrosis factor-alpha (TNF-α) in estrogen deficiency-induced bone loss *ex vivo*^3^. In this study, we further uncovered that lack of miR-21 in mice blocked osteopenia induced by OVX and during aging, suggesting protection of bone mass by miR-21 deficiency in pathological states. Our results also indicate the potential therapeutic effects of miR-21 inhibition on osteoporosis, which may be of clinical significance to investigate in future studies. We additionally demonstrated that serological miR-21 up-regulation was directly correlated with reduced BMD in human samples. Seeliger *et al*. also documented that expression of circulating and bone tissue miR-21 elevated in patients with osteoporotic fractures^27^. These findings highlighted potential values of miR-21 as both a therapeutic target and a biomarker for osteoporosis. For now, the source of serological miR-21 and its links with bone cells are still unknown, although we have detected the highest expression level of miR-21 in bone marrow among various tissues (data not shown). Our findings pave an avenue for further mechanistic researches on osteoclast-specific loss-of-function of miR-21 and miR-21 overexpression in skeletal disorders.

Interestingly, in the present study, we discovered *ex vivo* that miR-21 inhibited colony formation and proliferation of BMMSCs, while we confirmed its promotion on osteogenesis. Similarly, in mesenchymal stem cells derived from fetal amniotic fluid and adult adipose, overexpression of miR-21 *in vitro* suppressed proliferation while enhancing the differentiation potential^28^,^29^,^30^. The discrepancy of miR-21 effects in different functional aspects of BMMSCs lead to maintained bone formation and osteoblastogenesis in miR-21^−/−^ mice, as shown by our findings. Previous studies have also revealed that miR-23a has limited roles in bone formation *in vivo*^31^, in spite of its involvement in osteogenic differentiation *in vitro*^32^. Another important finding of the present study is that miR-21 reduced RANKL and induced OPG secretion physiologically, but these effects were not observed in pathological states. In multiple myeloma-derived BMMSCs, it has recently been reported that miR-21 directly targeted to reduce OPG and indirectly promoted RANKL by targeting the signal transducer and activator of transcription 3 (STAT3) pathway, suggesting inhibition of osteoclastogenesis by miR-21^10^. During bone mass accrual physiologically, we discovered in osteoblasts that miR-21 targeted Spry1 to regulate ERK signaling, thus suppressing RANKL and elevating OPG, indicating promotion of osteoclastogenesis by miR-21. The discrepancy of miR-21 effects on RANKL and OPG might be attributed to the distinguished physiological or pathological states, mature or progenitor cells, and *in vivo* or *in vitro* differences that leads to activation of different functional targets and signaling pathways. For example, the miR-21-Spry1-ERK axis was documented pivotal under stress^22^, and ERK signaling has been reported to regulate RANKL and OPG under mechanical force^21^, which is of great significance to maintain bone homeostasis *in vivo*^33^,^34^. Therefore, considering that the dynamic regulation of an individual microRNA (miR-21) on its potential targets may vary depending on the experimental conditions/microenvironments, direct *in vivo* elucidation of its functional importance based on gene-manipulated animals is of necessity.

In summary, we discovered *in vivo* the prevention of bone loss in miR-21^−/−^ mice that was attributed to an inhibition of osteoclast function. Our findings clarified skeletal function of miR-21 and provided first *in vivo* evidence of a pro-osteoclastic microRNA that regulates bone mass accrual and contributes to the development of osteoporosis.

## Methods

### Animals

All experimental protocols were approved by the Fourth Military Medical University. All animal experiments conducted in this research were performed in accordance with the guidelines of the Fourth Military Medical University Intramural Animal Use and Care Committee and met the NIH guidelines for the care and use of laboratory animals. WT C57BL/6 mice and miR-21^−/−^ mice were sourced directly from the Jackson Laboratory. For embryonic experiments, WT (n = 6) and miR-21^−/−^ (n = 6) embryos were separated at E18. For skeletal phenotype analysis during bone mass accrual, WT (n = 6, 3 female and 3 male) and miR-21^−/−^ (n = 6, 3 female and 3 male) mice were sacrificed at 3-month old^15^. For skeletal phenotype analysis post estrogen deficiency, 2-month female WT and miR-21^−/−^ mice underwent either a bilateral OVX (n = 6/genotype) or a Sham (n = 6/genotype) operation by the dorsal approach under general anesthesia^3^. Mice were modeled for 1 month and sacrificed at 3-month old. For age-related skeletal phenotype analysis, WT (n = 6, 3 female and 3 male) and miR-21^−/−^ (n = 6, 3 female and 3 male) mice were sacrificed at 16-month old^13^. The mice were maintained with good ventilation and a 12-h light/dark cycle, and were kept feeding and drinking ad libitum.

### Micro-CT analysis

For trabecular and cortical bone mass evaluation in mice, a desktop micro-CT system (eXplore Locus SP, GE Healthcare, USA) was employed^35^,^36^,^37^. At sacrifice, the left femora were isolated and fixed overnight in 4% paraformaldehyde. The distal femoral metaphysis were scanned at a resolution of 8 μm, a voltage of 80 kV, and a current of 80 μA. The region of interest (ROI) of the trabecular bone was defined from 0.1 mm to 2.6 mm away from the epiphysis. Cortical ROI was defined in the midshaft, from 3.5 mm to 4.0 mm away from the epiphysis. Data were analyzed with the Micview V2.1.2 software. Quantification of trabecular bone was performed using parameters of bone volume per tissue volume (BV/TV), BMD, trabecular thickness (Tb.Th), trabecular number (Tb.N), and trabecular separation (Tb.Sp)^3^. Quantification of cortical bone was performed using parameters of cortical thickness (Ct.Th) and cortical area fraction (Ct.Ar/Tt.Ar)^38^.

### Bone histology and histomorphometry

Embryos at E18 were separated, photographed by a stereomicroscope (SZX9, Olympus, Japan), and measured for body length. For whole-mount skeletal mineralization examination, skeletons were dissected, fixed in 95% ethanol for 5 days, placed in acetone for 2 days, stained in 0.0005% alizarin red solution (Sigma-Aldrich, USA) at 37 °C for 3 days, cleared, and photographed by a stereomicroscope (SZX9, Olympus, Japan), according to standard protocols with minor modifications^15^,^39^. Quantification was performed using the ImageJ 1.47 software for percentages of mineralized area in the lumbar spines and digits, as stated^15^. For histological observations on cartilaginous and mineralized tissue in skeleton, after isolation of embryos, tibiae were dissected, fixed with 4% paraformaldehyde, and sagittally sectioned without decalcification (RM2125, Leica, Germany). Alcian blue (Sigma-Aldrich, USA) and von Kossa staining were performed using standard protocols^15^. Quantification was performed using the ImageJ 1.47 software from at least five microscopic fields.

For bone formation examination, double calcein labeling was performed according to previous studies with minor modifications^3^,^37^,^40^. At 16 day and 2 day prior to sacrifice, mice received double intraperitoneal injection of 20 mg/kg calcein (Sigma-Aldrich, USA). Calcein was dissolved at a concentration of 2 mg/ml in PBS supplemented with 1 mg/ml NaHCO_3_ (Sigma-Aldrich, USA), and was injected at 10 μl/g each time away from light. Necessary precautions were taken to ensure that the injected fluid was never accidentally placed in intestine, and that successful administration of double calcein labeling was accomplished in all mice. At sacrifice, the right femora were isolated, fixed in 80% ethanol, and embedded in methyl methacrylate without decalcification. The specimens were sagittally sectioned into 30-μm sections using a hard tissue slicing machine (SP1600, Leica, Germany) away from light. Both double-labeled and single-labeled cortical endosteum surfaces were evaluated by a fluorescence microscope (STP6000, Leica, Germany) with an excitation wavelength of 488 nm. Quantification was performed based on at least five photographs using the parameters of mineral apposition rate (MAR) and mineralized surface per bone surface (MS/BS). Bone formation rate (BFR) was calculated as MAR × MS/BS, according to standard methods^40^.

For osteoblast and osteoclast/bone resorption examination, toluidine blue and TRAP staining was respectively performed, as stated before^15^,^40^. At sacrifice, left tibiae were isolated, fixed with 4% paraformaldehyde, decalcified with 10% ethylene diamine tetraacetic acid (EDTA) (pH, 7.2–7.4), and embedded in paraffin. 5-μm sagittal serial sections of proximal metaphyses were prepared (RM2125, Leica, Germany). The sections were either stained by 1% toluidine blue (Sigma-Aldrich, USA) dissolved in PBS for 30 min, or underwent TRAP staining using a commercial kit according to the manufacturers’ instructions (Sigma-Aldrich, USA). Osteoblast quantification was determined using parameters of number of osteoblasts per bone surface (N.Ob/BS) and osteoblast surface per bone surface (Ob.S/BS). Similarly, osteoclast/bone resorption quantification was determined using parameters of number of osteoclasts per bone surface (N.Oc/BS) and osteoclast surface per bone surface (Oc.S/BS). Quantification was performed using the ImageJ 1.47 software from at least five microscopic fields.

### Immunohistochemistry

Left tibiae were also used for *in situ* detection of miR-21 targets in bone marrow, according to published methods^13^. Sections were deparaffinized, treated by 0.25% trypsin (MP Biomedicals, USA) for 30 min at 37 °C for antigen retrieval, washed, and treated with 3% hydrogen peroxide for 20 min at 37 °C. Sections were blocked with 5% BSA (Sigma-Aldrich, USA) in PBS for 2 h in room temperature. Sections were then stained with either a rabbit anti-mouse PDCD4 primary antibody (Cell Signaling Technology, USA) or a rabbit anti-mouse p-c-fos primary antibody (Cell Signaling Technology, USA) overnight at 4 °C at both concentrations of 1:100, followed by a goat anti-rabbit secondary antibody (Cell Signaling Technology, USA) for 30 min in room temperature at a concentration of 1:200. Subsequently, an HRP-based Dako REAL^TM^ EnVision^TM^ Detection System (Dako, Denmark) was used to detect the immunoactivity, followed by counterstaining with hematoxylin (Sigma-Aldrich, USA). Negative control experiments were performed by omitting the primary antibodies. Quantification of number of positive stained cells over total area was performed using the ImageJ 1.47 software from at least five microscopic fields.

### ELISA

Collected murine serum underwent ELISA using murine ELISA kits according to the manufacturers’ instructions (R&D Systems, USA)^38^. Markers of bone resorption (CTX-1), bone formation (P1NP), RANKL and OPG were detected for their serological levels. Ratio of RANKL/OPG was calculated. RANKL and OPG were also detected for their concentrations in conditional media of osteoblasts.

### Human sample

Human sample collection and experiments were performed according to the Declaration of Helsinki in its newest version and published methods^27^,^41^. Serum specimens were obtained from patients with informed consent in Xijing Hospital that was pre-approved by the Fourth Military Medical University. Healthy donors and donors with postmenopausal osteoporosis were recruited. Exclusion criteria were tumors; diabetes; thyroid and parathyroid diseases; history of long-term hormone therapy; severe disorders in the digestive system; history of long-term immobilization; Cushing’s disease; history of liver and renal diseases; inflammation or auto-immune/inflammatory diseases; history of anti-osteoporotic medication; other metabolic endocrine diseases. The classification of osteoporosis was based on clinical dual-energy X-ray absorptiometry (DXA) for BMD of the lumbar spine. The characteristics of the included healthy donors (n = 9, female) and donors with postmenopausal osteoporosis (n = 9, female) were presented in Supplementary Table S2.

### Serum sample processing and microRNA extraction

For human serum samples, donors were fasted for food and liquids overnight. 5-mL whole blood was sampled via median cubital vein in the morning. For murine serum samples, before necropsy, the peripheral whole blood was collected from the retro-orbital venous plexus at 500 μL under general anesthesia. The serum were isolated by centrifuging at 3000 rpm 10 min followed by 12000 rpm 10 min at 4 °C. microRNAs from serum were extracted using the miRNeasy Serum/Plasma Kit according to the manufacture’s recommendations (Qiagen, Germany), followed by phenol-chloroform extraction, as described^27^. cDNA synthesis was performed using reverse transcription primers from the Bulge-loop^TM^ miRNA Primer Sets specific for murine and human miR-21 and RNU6 designed by RiboBio (Guangzhou, China), and PrimeScript^TM^ RT Reagent Kit (Takara, Japan)^42^,^43^.

### Statistical analysis

Murine data are represented as the mean ± standard errors of the mean. Statistical significance was evaluated by two-tailed Student’s t test for two-group comparison, and by one way analysis of variation (ANOVA) followed by Newman-Keuls post-hoc tests for multiple comparisons. Human data are given as box plots showing 5th, 50th and 95th percentiles, and minimum to maximum ranges. Two-tailed Mann-Whitney U test was used to determine the significance. Pearson’s test was used to determine the correlation between BMD and miR-21 expression. All the statistical tests were performed using SPSS 17.0 software. Values of *P* < 0.05 were considered as statistically significant.

## Additional Information

**How to cite this article:** Hu, C.-H. *et al*. miR-21 deficiency inhibits osteoclast function and prevents bone loss in mice. *Sci. Rep.* **7**, 43191; doi: 10.1038/srep43191 (2017).

**Publisher's note:** Springer Nature remains neutral with regard to jurisdictional claims in published maps and institutional affiliations.

## Supplementary Material

Supplementary Material
