# Author Correction: miR-21 deficiency inhibits osteoclast function and prevents bone loss in mice

**DOI:** 10.1038/s41598-022-25782-2

**Published:** 2022-12-14

**Authors:** Cheng-Hu Hu, Bing-Dong Sui, Fang-Ying Du, Yi Shuai, Chen-Xi Zheng, Pan Zhao, Xiao-Rui Yu, Yan Jin

**Affiliations:** 1 Department of Biochemistry and Molecular Biology, School of Basic Medical Sciences, Xi’an Jiaotong University Health Science Center, Xi’an, 710061 Shaanxi China; 2 State Key Laboratory of Military Stomatology & National Clinical Research Center for Oral Diseases & Shaanxi International Joint Research Center for Oral Diseases, Center for Tissue Engineering, Fourth Military Medical University, Xi’an, 710032 Shaanxi China; 3 Xi’an Institute of Tissue Engineering and Regenerative Medicine, Xi’an, 710032 Shaanxi China

Correction to: *Scientific Reports* 10.1038/srep43191, published online 27 February 2017

This Article contains errors in Figure 5 and Figure 7.

In Figure 5i in the “M, miR-21^−/−^ + PDCD4” group shows partial overlap with Figure 5e, the “M, WT” group. The corrected Figure 5 and its accompanying legend appears below.

Additionally, in Figure 7d, the “WT, aged” group overlaps with Figure 6d, the “WT, OVX” group. The corrected Figure 7, and its accompanying legend appears below.

**Figure 5:**
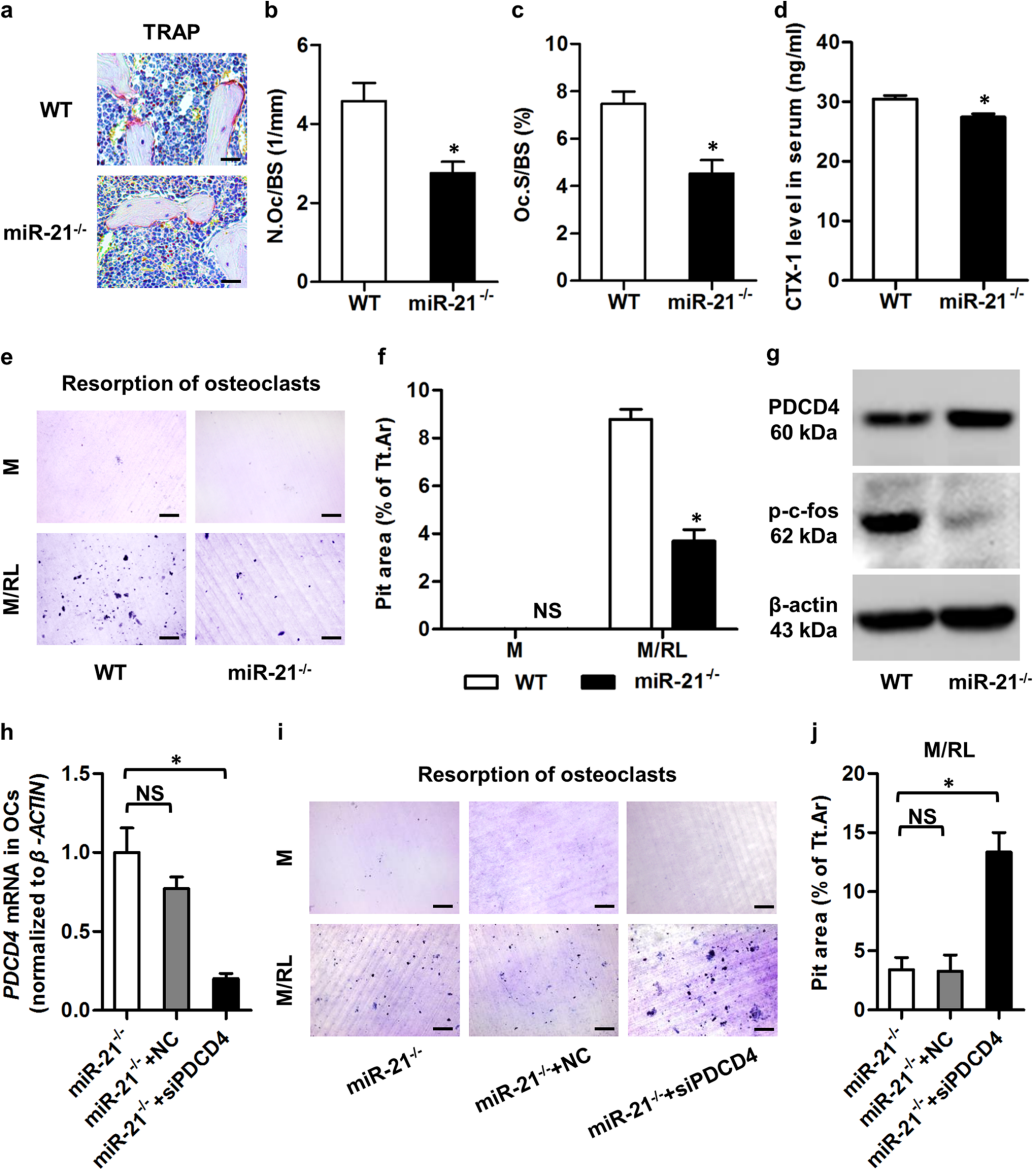
miR-21 promotes bone resorption in vivo and controls osteoclastogenesis by targeting programmed cell death 4 (PDCD4). (**a**) Tartrate resistant acid phosphotase (TRAP) staining of the trabecular bone in histological sections of 3-month WT and miR-21^−/−^ mice. Tibiae were decalcified, embedded in paraffin, sectioned, and stained for TRAP. Bars: 25 μm. (**b,c**) Corresponding parameters showed inhibited osteoclastogenesis and bone resorption in miR-21^−/−^ mice. N.Oc/BS, number of osteoclasts per bone surface (**b**). Oc.S/BS, osteoclast surface per bone surface (**c**). (**d**) Enzyme-linked immunosorbent assay (ELISA) detection of the serum bone resorption marker of 3-month WT and miR-21^−/−^ mice. miR-21 deficiency inhibited the bone resorption rate. CTX-1, cross linked C-telopeptide of type 1 collagen. (**e,f**) Representative images (**e**) and the corresponding parameter (**f**) demonstrated that miR-21^−/−^ osteoclasts (OCs) generated declined resorption pits on dentine slices. Resorption pits were stained with toluidine blue. M, macrophage colony-stimulating factor (M-CSF). RL, receptor activator of nuclear factor κB ligand (RANKL). Tt.Ar, total area. Bars: 100 μm. (**g**) Western blot analysis of mature OCs derived from 3-month WT and miR-21^−/−^ mice. OCs were differentiated with M-CSF and RANKL. miR-21 deficiency promoted the PDCD4 protein level, a functional target of miR-21, which suppressed the phosphorylation level of c-fos. Cropped blots are displayed with only brightness adjusted equally across the entire images. (**h**) Quantitative real-time polymerase chain reaction (qRT-PCR) analysis of miR-21^−/−^ mature OCs demonstrated down-regulation of mRNA level of *PDCD4* by small interfering RNA. OCs were differentiated with M-CSF and RANKL. siPDCD4, small interfering RNA for PDCD4. NC, negative control of siPDCD4. (**i,j**) Representative images (**i**) and the corresponding parameter (**j**) demonstrated that down-regulation of PDCD4 rescued resorption capability of miR-21^−/−^ OCs on dentine slices. Resorption pits were stained with toluidine blue. Bars: 100 μm. Data represents mean ± standard errors of the mean. n = 6/genotype (**a–g**), n = 3/group (**h**) and n = 4/group (**i,j**). Statistical significance was evaluated by two-tailed Student’s t test for two-group comparison, and one way analysis of variation (ANOVA) with Newman-Keuls post-hoc tests for multiple comparisons. *P < 0.05. NS, not significant (*P* > 0.05).

**Figure 7:**
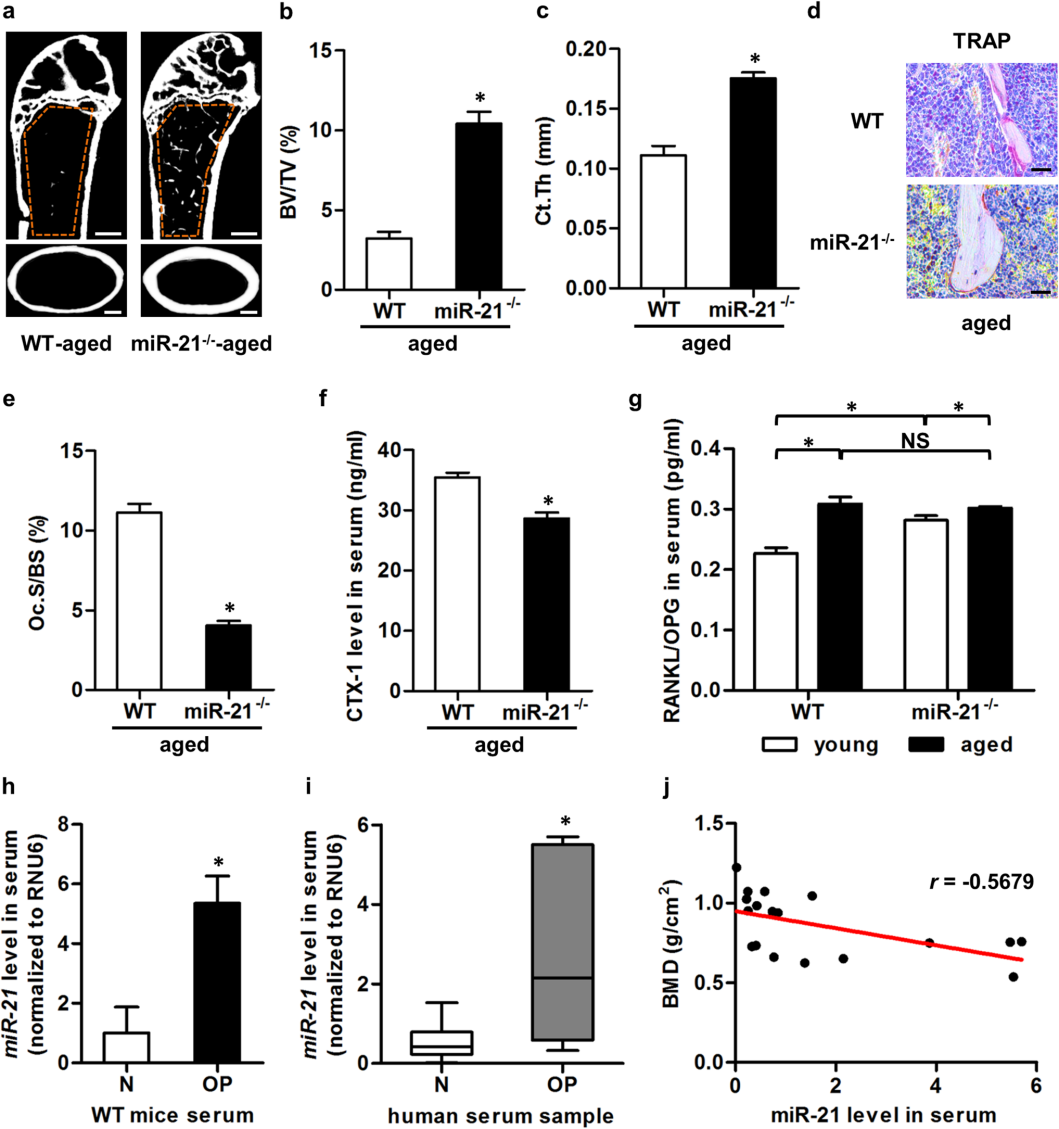
miR-21 contributes to age-related osteopenia and bone loss in human. (**a**) Representative micro-CT images demonstrating bone phenotypes of 16-month WT and miR-21^−/−^ mice. Orange frames indicate the region of interest analyzed for trabecular bone mass in the distal femoral metaphysis (up). Cortical bone mass was analyzed in the midshaft of femora (bottom). Bars: 500 μm. (**b**,**c**) Corresponding parameters showed that miR-21 deficiency prevented age-related trabecular (**b**) and cortical (**c**) bone loss. BV/TV, bone volume per tissue volume. Ct.Th, cortical thickness. (**d**) Tartrate resistant acid phosphotase (TRAP) staining of the trabecular bone of 16-month WT and miR-21^−/−^ mice. Tibiae were decalcified, embedded in paraffin, sectioned, and stained for TRAP. Bars: 25 μm. (**e**,**f**) The corresponding parameter of TRAP and the serum bone resorption marker detected by enzyme-linked immunosorbent assay (ELISA) showed that miR-21 deficiency blocked age-related osteoclastogenesis and bone resorption. Oc.S/BS, osteoclast surface per bone surface (**e**). CTX-1, cross linked C-telopeptide of type 1 collagen (**f**). (**g**) ELISA detection of serum ratio of receptor activator of nuclear factor κB ligand (RANKL) over osteoprotegerin (OPG). No significant difference was detected between 16-month WT and miR-21^−/−^ mice. (**h**) Quantitative real-time polymerase chain reaction (qRT-PCR) analysis demonstrated up-regulated mRNA level of miR-21 in serum of osteoporotic mice. N, normal. OP, osteopenia induced by ovariectomy (OVX). The above data represents mean ± standard errors of the mean. n = 6 per group of mice. Statistical significance was evaluated by two-tailed Student’s t test for two-group comparison, and by one way analysis of variation (ANOVA) followed by Newman-Keuls post-hoc tests for multiple comparisons. *P < 0.05. NS, not significant (*P* > 0.05). (**i**) In osteoporotic human samples, qRT-PCR analysis also detected up-regulated mRNA level of miR-21 in serum. N, healthy donor. OP, donor with postmenopausal osteoporosis. n = 9 per group. Results are given as box plots showing 5th, 50th and 95th percentiles, and minimum to maximum ranges. Two-tailed Mann–Whitney U test was used to determine the significance. *P < 0.05. (**j**) Bone mineral density (BMD) was inversely correlated with miR-21 in human serum. Pearson’s correlation: − 0.5679; *p* = 0.0140.

