# Supplementary material for: miR-21 deficiency inhibits osteoclast function and prevents bone loss in mice
Source: Sci Rep. 2017 Feb 27;7:43191. doi: 10.1038/srep43191 (PMC5327426; doi:10.1038/srep43191)
Supplement: Supplementary Material [file srep43191-s1.pdf]

miR-21 deficiency inhibits osteoclast function and prevents bone loss in mice

Cheng-Hu Hu[1, †], Bing-Dong Sui[2, †], Fang-Ying Du[1], Yi Shuai[2], Chen-Xi Zheng[2], Pan Zhao[3], Xiao-Rui Yu[1, *] & Yan Jin[2, 3, *]

[1]Department of Biochemistry and Molecular Biology, School of Basic Medical Sciences, Xi'an Jiaotong University Health Science Center, Xi'an, Shaanxi 710061, China.

[2]State Key Laboratory of Military Stomatology & National Clinical Research Center for Oral Diseases & Shaanxi International Joint Research Center for Oral Diseases, Center for Tissue Engineering, Fourth Military Medical University, Xi'an, Shaanxi 710032, China.

[3]Xi'an Institute of Tissue Engineering and Regenerative Medicine, Xi'an, Shaanxi 710032, China.

Supplementary Information: Supplementary Material including Supplementary Methods, Supplementary References, 5 Supplementary Figures and 3 Supplementary Tables is available.

Supplementary Methods

Genotyping. Genotyping for WT and miR-21$^{-/-}$ mice were performed according to published methods on microRNAs with minor modifications [1]. Briefly, genomic DNA was extracted from tail tips. For genotyping, polymerase chain reaction (PCR) was carried out using primers synthesized by Sangon Biotech (Shanghai, China). The sequences were directly sourced from the Jackson Laboratory. The primer for WT was: 5'-TTG CTT TAA ACC CTG CCT GAG CAC-3'. The primer for mutant miR-21 was: 5'-ACT TCC ATT TGT CAC GTC CTG CAC-3'. The PCR products were separated on an agarose gel. WT mice were identified with a band at 262 base pair (bp). miR-21$^{-/-}$ mice were identified with a band at 500 bp.

Body composition analysis. WT (n=6, 3 female and 3 male) and miR-21$^{-/-}$ (n=6, 3 female and 3 male) mice were sacrificed at 6-month old and measured for body weight. Organs/tissues of inguinal fat, quadriceps, heart, lung, liver, stomach, spleen, pancreas, kidney, bladder and gonad were respectively isolated and weighed. Each measure was repeated for at least three times.

Culture and analysis of murine bone marrow mesenchymal stem cells (BMMSCs). Isolation and culture of murine BMMSCs were as previously described [2,3]. Briefly, murine bone marrow cells were harvested from right tibiae and bilateral humeri and were seeded into 9-cm culture dishes. After incubation for 24 hours, non-adherent cells were removed and collected for osteoclast differentiation. The adherent cells were cultured with alpha-minimum

essential medium (α-MEM) (Invitrogen, USA) supplemented with 20% fetal bovine serum (FBS) (Invitrogen, USA), 2 mM L-glutamine (Invitrogen, USA), 100 U/ml penicillin (Invitrogen, USA), and 100 g/ml streptomycin (Invitrogen, USA) in a humidified atmosphere of 5% CO_2 at 37℃. The media were changed every 2 days. At confluence, BMMSCs were passaged with 0.25% trypsin (MP Biomedicals, USA). Approximately 1.2~2.4 × 10^6 1st-passaged BMMSCs could be obtained from a single mouse.

To induce osteogenic differentiation, BMMSCs at the 1st passage were cultured in osteogenic inducing media containing 100 μg/ml ascorbic acid (MP Biomedicals, USA), 2 mM β-glycerophosphate (Sigma-Aldrich, USA) and 10 nM dexamethasone (Sigma-Aldrich, USA). Cells were plated at 2 × 10^5 cells/well in 12-well plates. The media were changed every 3 days. After induction for 7 days, alkaline phosphatase (ALP) staining was performed to determine ALP activity in osteogenesis [2]. After induction for 14 days, alizarin red staining was performed to determine the mineralization in osteogenesis [1-3], and total RNA was collected by direct addition of Trizol Reagent (Takara, Tokyo, Japan) to replicate wells. The quantitative parameter of the percentage of mineralized area was determined with the ImageJ 1.47 software. RNA was further purified by phenol-chloroform extraction and reversed transcribed for cDNA synthesis, as described [3,4].

Colony forming efficiency (CFE) assay of BMMSCs was conducted as stated before [5]. After treated with ACK lysis buffer (Lonza, Switzerland) to remove red blood cells, murine primary bone marrow cells were plated in 5-cm culture dishes at a density of 1 × 10^5 cells/cm^2 and cultured. The formation of colonies was evaluated after 14 days of culture. The colonies were fixed with 4% paraformaldehyde for 30 min and stained with crystal violet for

5 min. Colonies with over 50 cells were counted using the ImageJ 1.47 software.

For proliferation analysis, BMMSCs at the 1st passage were plated at 2×10^3 cells/well in 96-well plates. At the same time in Day-1, 4, 7 and 10 (3 wells per time point), cells were incubated with 20-μl 5 mg/ml methyl thiazolyl tetrazolium (MTT) (MP Biomedicals, USA) for 4 h. The precipitates were extracted with 180-μl DMSO and the cell viability was measured at the optical density (OD) of 490 nm.

Culture and analysis of murine osteoblasts. Osteoblasts from adult murine long bone were isolated and cultured according to the published protocol [6]. Briefly, long bones without bone marrow were harvested, cut into pieces, washed with PBS, and incubated with collagenase II shaking at 37℃ for 2 h. The bone pieces were then rinsed with PBS, plated, and cultured with α-MEM (Invitrogen, USA) supplemented with 20% FBS (Invitrogen, USA), 2 mM L-glutamine (Invitrogen, USA), 100 U/ml penicillin (Invitrogen, USA), and 100 g/ml streptomycin (Invitrogen, USA) in a humidified atmosphere of 5% CO_2 at 37℃. The media were changed every 3 days. At confluence, primary osteoblasts were passaged with 0.25% trypsin (MP Biomedicals, USA).

For receptor activator of nuclear factor κB ligand (RANKL) and osteoprotegerin (OPG) examinations, osteoblasts at the 1st passage were plated at 2×10^5 cells/well in 12-well plates and cultured in 500-μl normal media for 24 h. siRNA transfections were performed as stated below, the media were then changed, and cells were then cultured in normal media for 24 h. For extracellular signal-regulated kinase (ERK) signaling inhibition, the pharmacological inhibitor PD98059 was added into the culture media at a concentration of 10 μM [7,8] for 24 h.

The conditional media were collected by centrifuging at 3000 rpm 10 min at 4℃ followed by 12000 rpm 10 min at 4℃ [9]. Total RNA was collected by direct addition of Trizol Reagent (Takara, Tokyo, Japan) to the culture plate. RNA was further purified by phenol-chloroform extraction and reversed transcribed for cDNA synthesis, as described [3,4].

Culture and analysis of murine osteoclasts. Osteoclasts were cultured and differentiated according to standard protocols [4,10]. Briefly, after 24-h incubation of total bone marrow cells, nonadherent cells were collected, seeded at 1×10^6 cells/well in 24-well plates, and cultured with 20 ng/ml macrophage colony-stimulating factor (M-CSF) in α-MEM (Invitrogen, USA) supplemented with 10% FBS (Invitrogen, USA), 2 mM L-glutamine (Invitrogen, USA), 100 U/ml penicillin (Invitrogen, USA), and 100 g/ml streptomycin (Invitrogen, USA) in a humidified atmosphere of 5% CO_2 at 37℃. After 3 days, the adherent cells were used as bone marrow macrophages (BMMs). BMMs were further cultured with 20 ng/ml M-CSF in the presence or absence of 50 ng/ml RANKL. siRNA transfections were performed as stated below. Tartrate resistant acid phosphotase (TRAP) staining was performed to determine mature osteoclasts, and $TRAP^+$ multinucleated cells with over 3 nuclei were identified as mature osteoclasts [4]. Total RNA of mature osteoclasts was collected by direct addition of Trizol Reagent (Takara, Tokyo, Japan) to replicate wells. RNA was further purified by phenol-chloroform extraction and reversed transcribed for cDNA synthesis, as described [3,4].

For resorption assay, as reported [11,12], dentine slices with 4-mm length, 4-mm width and 200-μm thick were prepared by cutting human premolars with a low-speed cutting machine (SYJ-150, Kejing Instrument, China). Human healthy premolars extracted for orthodontic

needs were kindly provided by the Department of Orthodontics, School of Stomatology, the Fourth Military Medical University. Dentine slices were cleaned by ultrasonication in distilled water, sterilized using 75% ethanol, and plated in 24-well plates overnight under ultraviolet light. BMMs were harvested, seeded on dentine slices, and further cultured with 20 ng/ml M-CSF in the presence or absence of 50 ng/ml RANKL. siRNA transfections were performed as stated below. At the end of the test, cells were removed from dentine slices by wiping the surfaces, and the resorption pits were stained with 1 μg/ml toluidine blue [13]. Percentages of pit area over total area were determined using the ImageJ 1.47 software.

siRNA transfection. siRNA transfections were performed according to our previous protocol [14]. siRNAs for Sprouty 1 (Spry1) and programmed cell death 4 (PDCD4) and their respective negative controls (NCs) were purchased from RiboBio (Guangzhou, China). After seeding of osteoblasts and BMMs and at 50%-70% confluent, the diluted siRNAs or NCs were combined with the supplied transfection reagent and added to each well according to the standard protocol. The siRNAs or NCs were transfected at final concentrations of 100 nM. Cells were then incubated for 24 hours before further assays. Transfection efficacy tests were also performed after 24 h incubation, by direct addition of Trizol Reagent (Takara, Tokyo, Japan) to replicate wells. RNA was further purified by phenol-chloroform extraction and reversed transcribed for cDNA synthesis, as described [3,4].

Quantitative real-time polymerase chain reaction (qRT-PCR) analysis. qRT-PCR analysis of microRNAs and mRNAs were according to previous studies [3,15]. For microRNAs,

qRT-PCR primers from the Bulge-loop™ miRNA qRT-PCR Primer Sets were designed by RiboBio (Guangzhou, China). For mRNAs, RNA was further purified by phenol-chloroform extraction and reversed transcribed for cDNA synthesis, and the primer sequences were listed in Supplementary Table S3. qRT-PCR were performed using the SYBR Premix Ex Taq II Kit (Takara, Japan) and detected by a Real-Time System (CFX96, Bio-Rad, USA). The relative expression level of each gene was obtained by the cycle number after normalizing against *RNU6* (for *miR-21*) and *ACTIN* (for mRNAs) abundances using the $2^{-\Delta\Delta CT}$ method.

Western blotting. Western blotting was performed as previously described [3]. Whole-cell lysates of BMMSCs underwent osteogenic induction for 14 days, osteoblasts after siSPRY1 transfection for 24 h, and mature osteoclasts were prepared using the Cell Lysis Buffer (Beyotime, China). Proteins were extracted, loaded on sodium dodecyl sulfate-polyacrylamide gels, transferred to polyvinylidene fluoride membranes (Millipore, USA), and blocked with 5% bovine serum albumin (BSA) (Sigma-Aldrich, USA) in PBST (PBS with 0.1% Tween) for 2 h in room temperature. The membranes were incubated overnight at 4℃ with the following primary antibodies: a rabbit anti-mouse primary antibody at a concentration of 1:1000 for Runt-related transcription factor 2 (Runx2) (Cell Signaling Technology, USA) in BMMSCs; a rabbit anti-mouse primary antibody at a concentration of 1:1000 for Spry1 (Santa Cruz Biotechnology, USA) in BMMSCs; a rabbit anti-mouse primary antibody at a concentration of 1:1000 for ERK1/2 (Cell Signaling Technology, USA) in osteoblasts; a rabbit anti-mouse primary antibody at a concentration of 1:1000 for p-ERK1/2 (Cell Signaling Technology, USA) in osteoblasts; a rabbit anti-mouse

primary antibody at a concentration of 1:1000 for PDCD4 (Cell Signaling Technology, USA) in osteoclasts; a rabbit anti-mouse primary antibody at a concentration of 1:1000 for p-c-fos (Cell Signaling Technology, USA) in osteoclasts; a rabbit anti-mouse primary antibody at a concentration of 1:4000 for β-tubulin (Abcam, USA) in osteoblasts; and a mouse anti-mouse primary antibody at a concentration of 1:4000 for β-actin (Abcam, USA) in BMMSCs and osteoclasts. The membranes were then incubated with peroxidase-conjugated secondary antibodies (Boster, China) for 1 h in room temperature. The blotted bands were visualized using an enhanced chemiluminescence Kit (Amersham Biosciences, USA) and a gel imaging system (5500, Tanon, China). Only brightness was adjusted equally across the entire images.

Supplementary References

1. Li, C.J., *et al.* MicroRNA-188 regulates age-related switch between osteoblast and adipocyte differentiation. *J. Clin. Invest.* **125**, 1509-1522 (2015).

2. Yang, N., *et al.* Tumor necrosis factor alpha suppresses the mesenchymal stem cell osteogenesis promoter miR-21 in estrogen deficiency-induced osteoporosis. *J. Bone. Miner. Res.* **28**, 559-573 (2013).

3. Liao, L., *et al.* Redundant miR-3077-5p and miR-705 mediate the shift of mesenchymal stem cell lineage commitment to adipocyte in osteoporosis bone marrow. *Cell. Death. Dis.* **4**, e600 (2013).

4. Sugatani, T., Vacher, J. & Hruska, K.A. A microRNA expression signature of osteoclastogenesis. *Blood.* **117**, 3648-3657 (2011).

5. Wu, X., *et al.* Inhibition of Sca-1-positive skeletal stem cell recruitment by alendronate blunts the anabolic effects of parathyroid hormone on bone remodeling. *Cell. Stem. Cell.* **7**, 571-580 (2010).

6. Bakker, A.D. & Klein-Nulend, J. Osteoblast isolation from murine calvaria and long bones. *Methods. Mol. Biol.* **816**, 19-29 (2012).

7. Li, S., *et al.* Connexin 43 and ERK regulate tension-induced signal transduction in human periodontal ligament fibroblasts. *J. Orthop. Res.* **33**, 1008-1014 (2015).

8. Fukushima, H., Jimi, E., Okamoto, F., Motokawa, W. & Okabe, K. IL-1-induced receptor activator of NF-kappa B ligand in human periodontal ligament cells involves ERK-dependent PGE2 production. *Bone.* **36**, 267-275 (2005).

9. Kobayashi, A., *et al.* Bone morphogenetic protein 7 in dormancy and metastasis of

prostate cancer stem-like cells in bone. *J. Exp. Med.* **208**, 2641-2655 (2011).

10. Takeshita, S., Kaji, K. & Kudo, A. Identification and characterization of the new osteoclast progenitor with macrophage phenotypes being able to differentiate into mature osteoclasts. *J. Bone. Miner. Res.* **15**, 1477-1488 (2000).

11. Tamura, T., *et al.* New resorption assay with mouse osteoclast-like multinucleated cells formed in vitro. *J. Bone. Miner. Res.* **8**, 953-960 (1993).

12. Elefteriou, F., *et al.* Leptin regulation of bone resorption by the sympathetic nervous system and CART. *Nature.* **434**, 514-520 (2005).

13. Noh, A.L., Park, H., Zheng, T., Ha, H.I. & Yim, M. L-type Ca(2+) channel agonist inhibits RANKL-induced osteoclast formation via NFATc1 down-regulation. *Life. Sci.* **89**, 159-164 (2011).

14. Liao, L., *et al.* TNF-alpha inhibits FoxO1 by upregulating miR-705 to aggravate oxidative damage in bone marrow-derived mesenchymal stem cells during osteoporosis. *Stem. Cells.* **34**, 1054-1067 (2016).

15. Liang, D., *et al.* A human herpesvirus miRNA attenuates interferon signaling and contributes to maintenance of viral latency by targeting IKKepsilon. *Cell. Res.* **21**, 793-806 (2011).

Supplementary Figures

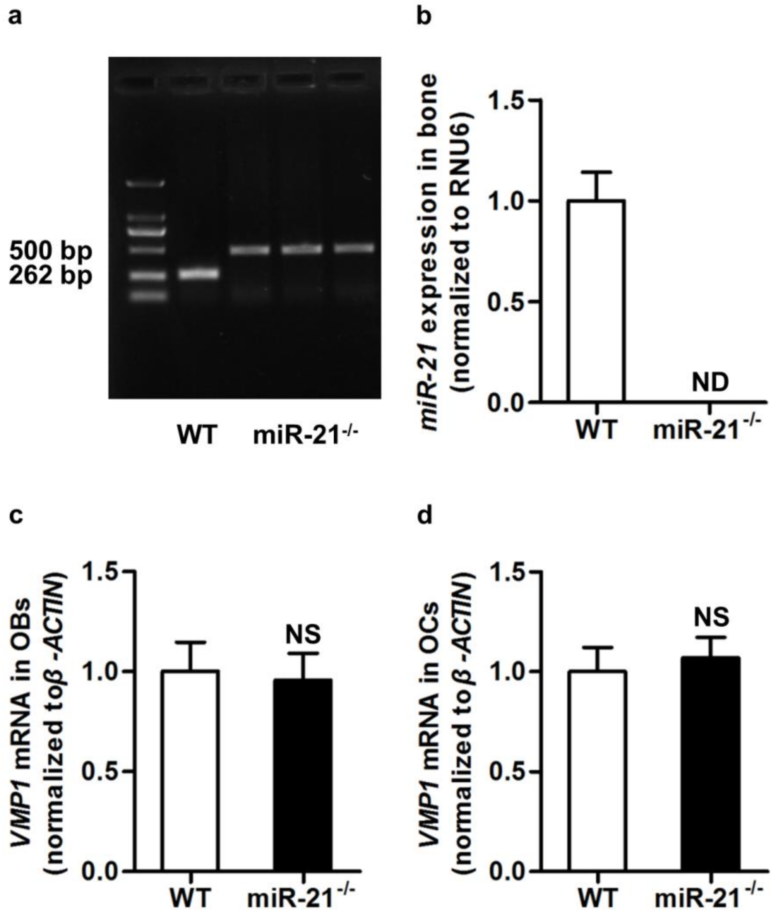

Supplementary Figure S1. Confirmation of the miR-21-deficient status in miR-21$^{-/-}$ mice. (**a**) Polymerase chain reaction (PCR)-based genotyping analysis demonstrated general knock out of miR-21. WT mice were identified with a band at 262 bp. miR-21$^{-/-}$ mice were identified with a band at 500 bp. (**b**) Quantitative real-time polymerase chain reaction (qRT-PCR) analysis demonstrated deficient mRNA expression of miR-21 in bone from miR-21$^{-/-}$ mice. (**c, d**) qRT-PCR analysis demonstrated comparable mRNA expression levels of *Vacuole membrane protein-1* (*VMP1*) in osteoblasts (OBs) (**c**) and osteoclasts (OCs) (**d**) derived from WT and miR-21$^{-/-}$ mice. The gene of *VMP1*, also known as *Transmembrane protein 49* (*TMEM49*), overlaps with the gene encoding miR-21. Data indicated that the neighborhood of miR-21 gene loci is not affected by miR-21 knockout. Data represents mean

± standard errors of the mean. n=3/genotype. Statistical significance was evaluated by two-tailed Student's t test. ND, not detected. NS, not significant ($P > 0.05$).

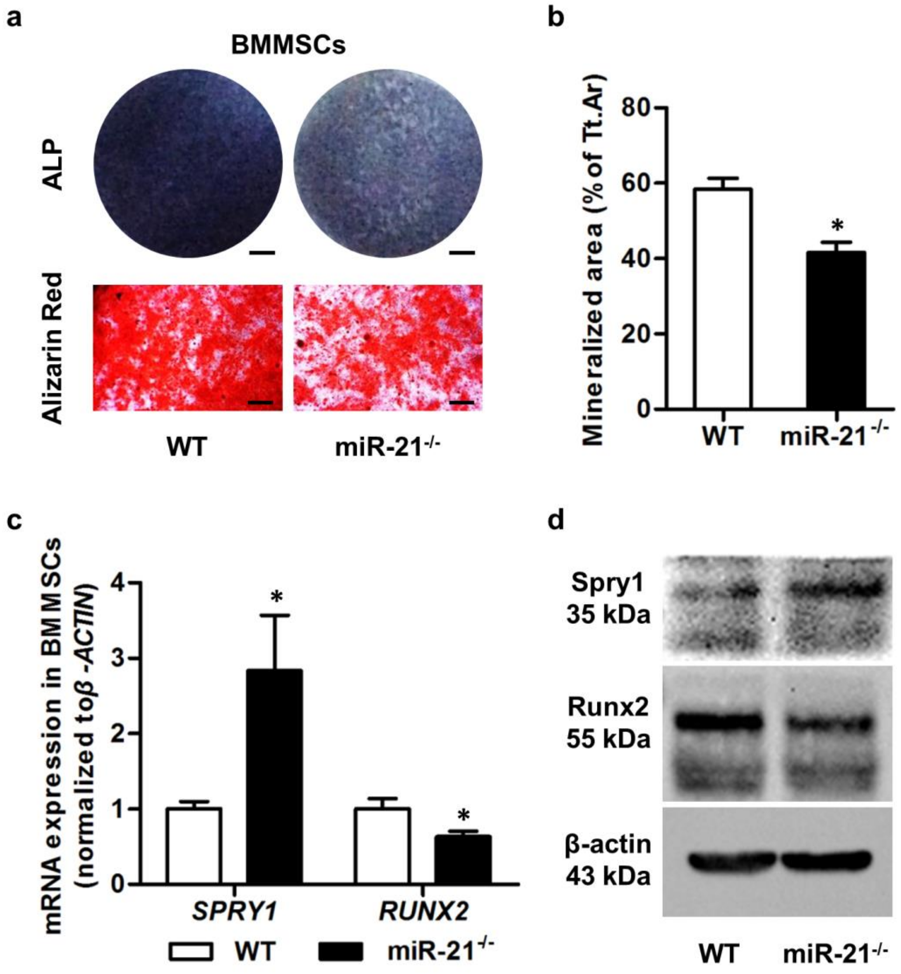

Supplementary Figure S2. Declined osteogenic differentiation in miR-21$^{-/-}$ bone marrow mesenchymal stem cells (BMMSCs) through up-regulation of Sprouty 1 (Spry1). (**a**) Alkaline phosphatase (ALP) staining (top) and alizarin red staining (bottom) showed that miR-21$^{-/-}$ BMMSCs demonstrated reduced osteogenic potential. 1st passaged BMMSCs isolated from 3-month WT and miR-21$^{-/-}$ mice were seeded at 2×10^5 cells/well in 12-well plates and underwent osteogenic induction. ALP staining was performed at Day-7. Alizarin red staining was performed at Day-14. Bars (top): 2 mm; Bars (bottom): 100 μm. (**b**) The corresponding parameter of alizarin red staining showed declined mineralization by miR-21$^{-/-}$ BMMSCs. Tt.Ar, total area. (**c**) Quantitative real-time polymerase chain reaction (qRT-PCR) analysis demonstrated up-regulated mRNA level of *SPRY1* in BMMSCs from 3-month WT

and miR-21$^{-/-}$ mice. The osteogenic marker gene Runt-related transcription factor 2 (*RUNX2*) mRNA expression was down-regulated in miR-21$^{-/-}$ BMMSCs. BMMSCs underwent osteogenic differentiation for 14 days. (**d**) Western blot analysis of BMMSCs derived from 3-month WT and miR-21$^{-/-}$ mice. BMMSCs underwent osteogenic differentiation for 14 days. miR-21 deficiency promoted Spry1 expression and inhibited Runx2 expression. Cropped blots are displayed with only brightness adjusted equally across the entire images. Data represents mean ± standard errors of the mean. n=6/genotype. Statistical significance was evaluated by two-tailed Student's t test. *, $P < 0.05$. NS, not significant ($P > 0.05$).

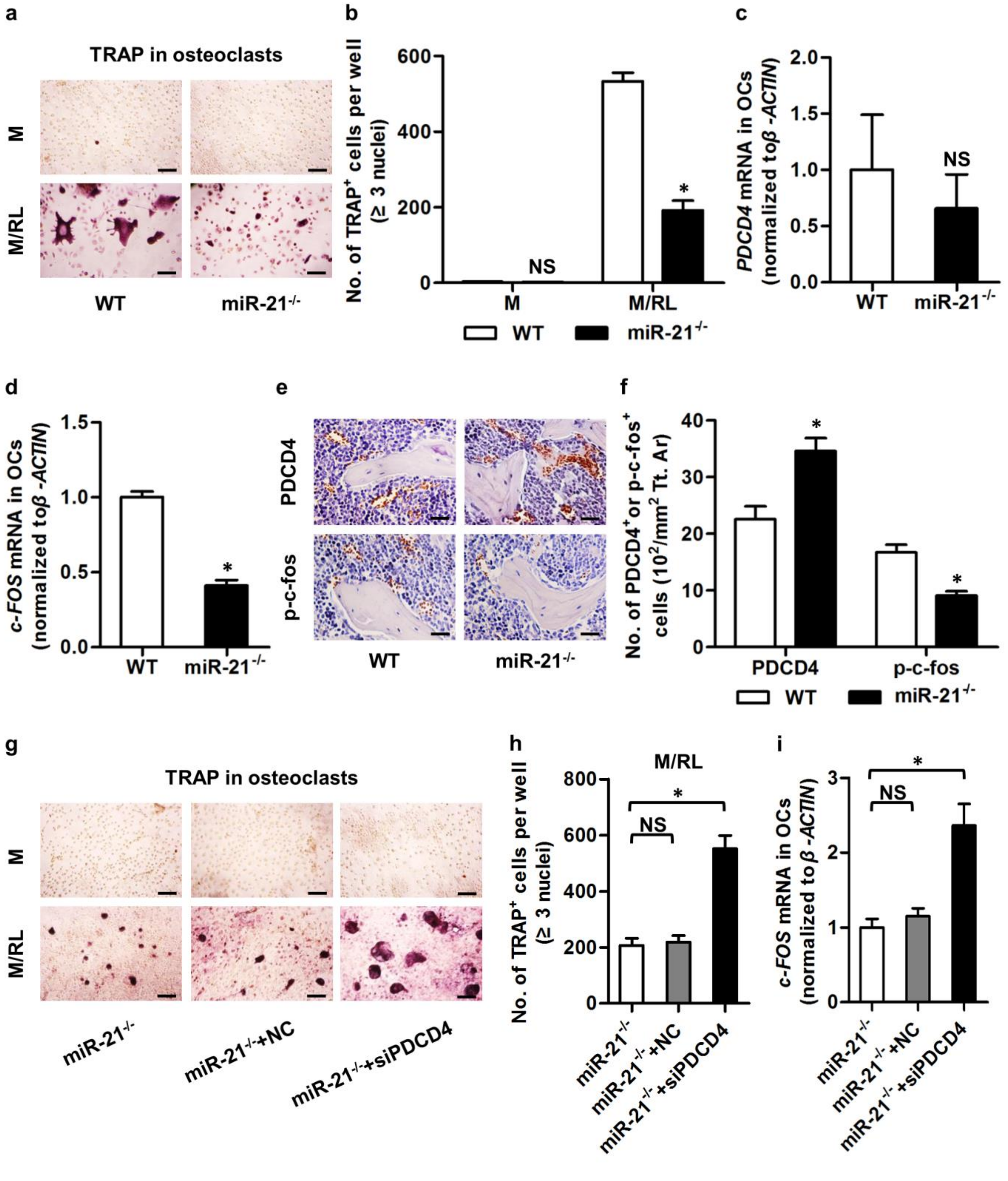

Supplementary Figure S3. miR-21 supports osteoclastogenesis by targeting programmed cell death 4 (PDCD4) in osteoclasts (OCs). (**a, b**) Representative images (**a**) and the corresponding parameter (**b**) demonstrated that miR-21$^{-/-}$ bone macrophages (BMMs) generated declined numbers of mature OCs. Cells were stained with tartrate resistant acid phosphotase (TRAP), and TRAP^{+} cells with over 3 nuclei were identified as OCs. M,

macrophage colony-stimulating factor (M-CSF). RL, receptor activator of nuclear factor κB ligand (RANKL). Bars: 100 μm. (**c, d**) Quantitative real-time polymerase chain reaction (qRT-PCR) analysis of mature OCs derived from 3-month WT and miR-21$^{-/-}$ mice. OCs were differentiated with M-CSF and RANKL from BMMs. miR-21 deficiency did not influence the PDCD4 mRNA level, a functional target of miR-21 (**c**). The mRNA level of c-fos, a critical transcription factor for osteoclastogenesis, was suppressed (**d**). (**e, f**) Representative immunohistochemistry images (**e**) and corresponding parameters (**f**) demonstrated that miR-21 deficiency promoted PDCD4^{+} cells and inhibited p-c-fos^{+} cells surrounding trabecular bone, suggesting that miR-21 supported osteoclastogenesis in vivo through targeting PDCD4 to regulate c-fos. Tt.Ar, total area. Bars: 25 μm. (**g, h**) Representative images (**g**) and the corresponding parameter (**h**) demonstrated that down-regulation of PDCD4 in miR-21$^{-/-}$ BMMs rescued differentiation of OCs. Cells were stained with TRAP, and TRAP^{+} cells with over 3 nuclei were identified as OCs. M, M-CSF. RL, RANKL. siPDCD4, small interfering RNA for PDCD4. NC, negative control of siPDCD4. Bars: 100 μm. (**i**) qRT-PCR analysis of miR-21$^{-/-}$ mature OCs demonstrated rescue of mRNA level of *c-FOS* by siPDCD4. OCs were differentiated with M-CSF and RANKL from miR-21$^{-/-}$ BMMs. Data represents mean ± standard errors of the mean. n=6/genotype (**a-f**), n=4/group (**g, h**) and n=3/group (**i**). Statistical significance was evaluated by two-tailed Student's t test for two-group comparison, and one way analysis of variation (ANOVA) with Newman-Keuls post-hoc tests for multiple comparisons. *, $P < 0.05$. NS, not significant ($P > 0.05$).

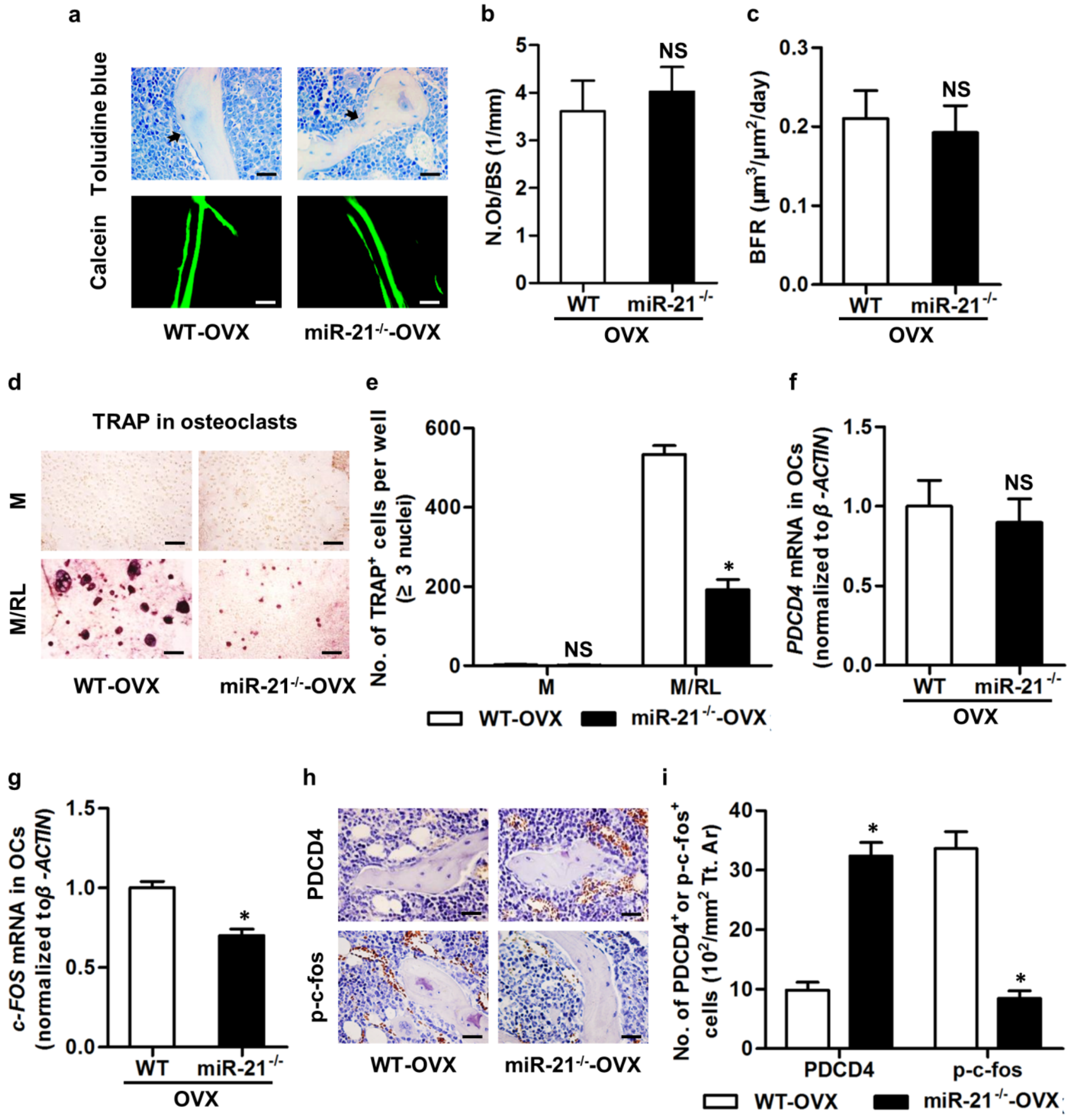

Supplementary Figure S4. Impaired bone formation and blocked osteoclastogenesis through programmed cell death 4 (PDCD4) in miR-21-deficient ovariectomized mice. (a) Toluidine blue staining (top) and calcein labeling (bottom) in histological sections of WT and miR-21$^{-/-}$ mice at 1 month post ovariectomy (OVX). Mice accepted double intraperitoneal injection of 20 mg/kg calcein at 16 days and 2 days prior to sacrifice. After sacrifice, tibiae were decalcified, embedded in paraffin, sectioned, and stained for toluidine blue. Femora were embedded in methyl methacrylate without decalcification, sectioned, and observed by a

fluorescence microscope on the endosteum. Black arrows indicate osteoblasts analyzed on trabecular bone surfaces. Bars (top): 25 μm; Bars (bottom): 100 μm. (**b**) The corresponding parameter of toluidine blue staining showed comparable inhibition of osteoblastogenesis in ovariectomized WT and miR-21$^{-/-}$ mice. N.Ob/BS, number of osteoblasts per bone surface. (**c**) The corresponding parameter detected by calcein labeling showed comparable impairments of bone formation in ovariectomized WT and miR-21$^{-/-}$ mice. BFR, bone formation rate. (**d, e**) Representative images (**d**) and the corresponding parameter (**e**) demonstrated that miR-21 deficiency blocked OVX-induced osteoclast differentiation. Cells were stained with tartrate resistant acid phosphotase (TRAP), and TRAP$^+$ cells with over 3 nuclei were identified as osteoclasts (OCs). M, macrophage colony-stimulating factor (M-CSF). RL, receptor activator of nuclear factor κB ligand (RANKL). Bars: 100 μm. (**f, g**) qRT-PCR analysis of mature OCs derived from ovariectomized WT and miR-21$^{-/-}$ mice. OCs were differentiated with M-CSF and RANKL from bone marrow macrophages (BMMs). After OVX, miR-21 deficiency did not influence the PDCD4 mRNA level, a functional target of miR-21 (**f**). The mRNA level of c-fos, a critical transcription factor for osteoclastogenesis, was suppressed (**g**). (**h, i**) Representative immunohistochemistry images (**h**) and corresponding parameters (**i**) demonstrated that miR-21 deficiency promoted OVX-reduced PDCD4$^+$ cells and inhibited OVX-induced p-c-fos$^+$ cells surrounding trabecular bone, suggesting that miR-21 deficiency blocked OVX-induced osteoclastogenesis in vivo through targeting PDCD4 to regulate c-fos. Tt.Ar, total area. Bars: 25 μm. Data represents mean ± standard errors of the mean. n=6/genotype. Statistical significance was evaluated by two-tailed Student's t test. *, $P < 0.05$. NS, not significant ($P > 0.05$).

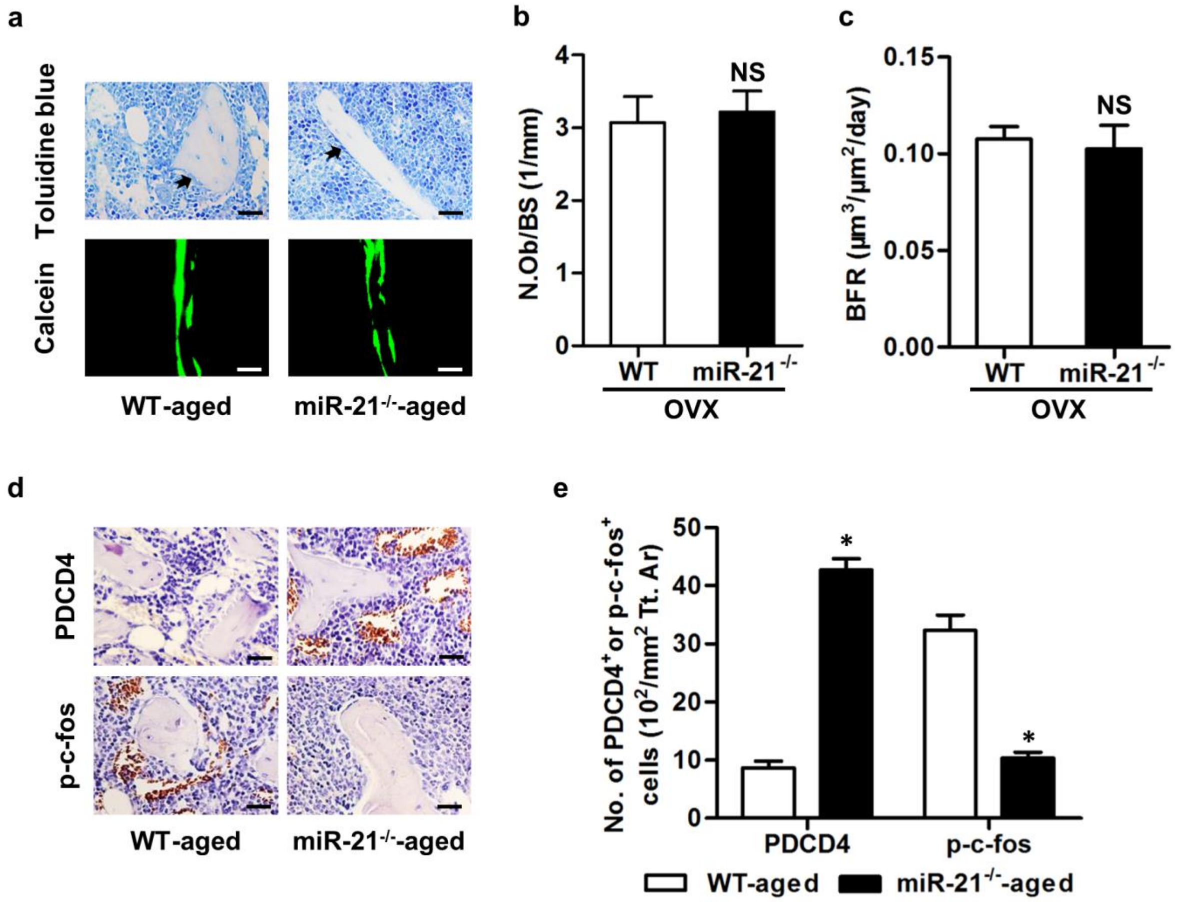

Supplementary Figure S5. Bone formation and target examination in miR-21-deficient aged mice. (**a**) Toluidine blue staining (top) and calcein labeling (bottom) in histological sections of 16-month WT and miR-21$^{-/-}$ mice. Mice accepted double intraperitoneal injection of 20 mg/kg calcein at 16 days and 2 days prior to sacrifice. After sacrifice, tibiae were decalcified, embedded in paraffin, sectioned, and stained for toluidine blue. Femora were embedded in methyl methacrylate without decalcification, sectioned, and observed by a fluorescence microscope on the endosteum. Black arrows indicate osteoblasts analyzed on trabecular bone surfaces. Bars (top): 25 μm; Bars (bottom): 100 μm. (**b**) The corresponding parameter of toluidine blue staining showed comparable inhibition of osteoblastogenesis in 16-month WT and miR-21$^{-/-}$ mice. N.Ob/BS, number of osteoblasts per bone surface. (**c**) The corresponding parameter detected by calcein labeling showed comparable impairments of bone formation in

16-month WT and miR-21$^{-/-}$ mice. BFR, bone formation rate. (**d, e**) Representative immunohistochemistry images (**d**) and corresponding parameters (**e**) demonstrated that miR-21 deficiency promoted age-reduced programmed cell death 4 (PDCD4)$^{+}$ cells and inhibited age-induced p-c-fos^{+} cells surrounding trabecular bone, suggesting that miR-21 deficiency blocked age-related osteoclastogenesis in vivo through targeting PDCD4 to regulate c-fos. Tt.Ar, total area. Bars: 25 μm. Data represents mean ± standard errors of the mean. n=6/genotype. Statistical significance was evaluated by two-tailed Student's t test. *, $P < 0.05$. NS, not significant ($P > 0.05$).

Supplementary Tables

	**WT-male**	**miR-21$^{-/-}$-male**	**WT-female**	**miR-21$^{-/-}$-female**
Body weight	28.00 ± 0.62	30.57 ± 1.00*	27.08 ± 0.48	28.98 ± 0.34$^{\#}$
Inguinal fat	0.33 ± 0.02	0.34 ± 0.02	0.30 ± 0.01	0.31 ± 0.01
Quadriceps	0.22 ± 0.01	0.22 ± 0.01	0.20 ± 0.01	0.20 ± 0.01
Heart	0.23 ± 0.02	0.24 ± 0.03	0.24 ± 0.02	0.24± 0.03
Lung	0.18 ± 0.01	0.18 ± 0.02	0.19 ± 0.01	0.22 ± 0.02
Liver	1.52 ± 0.10	1.54 ± 0.10	1.55 ± 0.14	1.49 ± 0.16
Stomach	0.84 ± 0.12	0.84 ± 0.08	0.73 ± 0.03	0.70 ± 0.12
Spleen	0.19 ± 0.02	0.19 ± 0.02	0.19 ± 0.01	0.19 ± 0.02
Pancreas	0.18 ± 0.02	0.17 ± 0.01	0.18 ± 0.01	0.17 ± 0.01
Kidney	0.16 ± 0.01	0.17 ± 0.00	0.15 ± 0.01	0.15 ± 0.01
Bladder	0.03 ± 0.00	0.03 ± 0.00	0.03 ± 0.00	0.03 ± 0.00
Gonad	0.13 ± 0.01	0.14 ± 0.02	0.00 ± 0.00	0.00 ± 0.00

Supplementary Table S1. Body composition of 6-month old WT and miR-21$^{-/-}$ mice. Data represent mean (g) ± standard errors of the mean (g). n=3 per group. $^{*}P < 0.05$ with the WT-male group; $^{\#}P < 0.05$ with the WT-female group.

**ID number**	**Gender**	**Age**	**BMD (g/cm^2)**	**T-score**
N-1	Female	46	0.939	-1
N-2	Female	47	0.951	0.9
N-3	Female	52	0.982	-0.6
N-4	Female	48	0.946	-0.9
N-5	Female	46	1.024	-0.2
N-6	Female	54	1.223	1.6
N-7	Female	52	1.071	0.2
N-8	Female	46	1.071	0.2
N-9	Female	54	1.045	0
OP-1	Female	55	0.749	-2.7
OP-2	Female	54	0.757	-2.6
OP-3	Female	50	0.727	-2.9
OP-4	Female	47	0.734	-2.8
OP-5	Female	63	0.661	-3.5
OP-6	Female	65	0.651	-3.6
OP-7	Female	63	0.536	-4.6
OP-8	Female	64	0.624	-3.8
OP-9	Female	63	0.754	-2.7

Supplementary Table S2. Characteristics of human serum donors. BMD, bone mineral density of the lumbar spine; N, healthy donor; OP, donor with osteoporosis (T-score < -2.5).

Gene	Primer sequences
β-ACTIN	Forward: 5’-CATCCGTAAAGACCTCTATGCCAAC-3’
	Reverse: 5’-ATGGAGCCACCGATCCACA-3’
VMP1	Forward: 5’-CCAAACCCCCTGTTTGACCT-3’
	Reverse: 5’-CACCATCTGCTCCACGATGT-3’
RUNX2	Forward: 5’-AAATGCCTCCGCTGTTATGAA-3’
	Reverse: 5’-AAGGTGAAACTCTTGCCTCGTC-3’
SPRY1	Forward: 5’-GGTGCCACTGCTACTATGGG-3’
	Reverse: 5’-GGTGCCACTGCTACTATGGG-3’
RANKL	Forward: 5’-TGTACTTTCGAGCGCAGATG-3’
	Reverse: 5’-CCACAATGTGTTGCAGTTCC-3’
OPG	Forward: 5’-CTGATGTATGCCCTCAAGCA-3’
	Reverse: 5’-AAACAGCCCAGTGACCATTC-3’
PDCD4	Forward: 5’-AGCGGTTAGAAGTGGAGTTGCTGT-3’
	Reverse: 5’-ACAAGGTGATTGACAGGCTGTTGC-3’
c-FOS	Forward: 5’- CCAAGCGGAGACAGATCAACTT-3’
	Reverse: 5’- TCCAGTTTTTCCTTCTCTTTCAGCAGA-3’

Supplementary Table S3. Primer sequences for mRNAs in the present study.
